# Loss of Endothelial YAP/TAZ Reduces the Size of Chronic Stroke Lesions and Alters the Endothelial Environment

**DOI:** 10.1161/JAHA.124.040079

**Published:** 2025-11-26

**Authors:** Ria Göttert, Majed Kikhia, Marie‐Louise Herzog, Wen Pan, Alexandra Klaus‐Bergmann, January Weiner, Dieter Beule, Samuel Knauss, Golo Kronenberg, Michael Potente, Holger Gerhardt, Matthias Endres, Karen Gertz

**Affiliations:** ^1^ Charité—Universitätsmedizin Berlin, Corporate Member of Freie Universität Berlin, Humboldt‐Universität zu Berlin, and Berlin Institute of Health, Klinik für Neurologie und Abteilung für Experimentelle Neurologie Berlin Germany; ^2^ DZHK (German Centre for Cardiovascular Research), Partner Site Berlin Germany; ^3^ Charité—Universitätsmedizin Berlin, Einstein Center for Neurosciences Berlin Germany; ^4^ Max Delbrück Center for Molecular Medicine in the Helmholtz Association (MDC) Berlin Germany; ^5^ Core Unit Bioinformatics, Berlin Institute of Health at Charité Berlin Germany; ^6^ Klinik für Psychiatrie, Psychotherapie und Psychosomatik, Psychiatrische Universitätsklinik Zürich Switzerland; ^7^ Angiogenesis & Metabolism Laboratory, Berlin Institute of Health at Charité—Universitätsmedizin Berlin Germany; ^8^ German Center for Mental Health (DZPG), Partner Site Berlin Germany; ^9^ German Center for Neurodegenerative Diseases (DZNE), Partner Site Berlin Germany

**Keywords:** endothelial cells, inflammation, ischemic stroke, neuroprotection, YAP/TAZ, Cerebrovascular Disease/Stroke, Ischemic Stroke, Neuroprotectants

## Abstract

**Background:**

Ischemic stroke remains a leading cause of morbidity and mortality worldwide, with limited treatment options available. Vascular dysfunction is a key pathomechanism, and brain endothelial cells (bECs) play a critical role in determining stroke outcomes. This study investigates the specific roles of YAP (yes‐associated protein 1) and TAZ (WW domain containing transcription regulator 1) in regulating bEC functions during stroke.

**Methods:**

Mice underwent 30‐minute middle cerebral artery occlusion (MCAo) followed by reperfusion to model ischemic stroke. TAZ reporter mice were used to track stroke‐induced subcellular changes in TAZ expression. Tamoxifen‐inducible endothelial‐specific *Yap*/*Taz* knockout and control mice were used to study YAP/TAZ’s role in bEC function post‐stroke. Stroke outcomes were measured by magnetic resonance imaging and NeuN (neuronal nuclei)‐associated lesion analysis. Properties of bECs were assessed via immunohistochemistry and RNA sequencing. Inflammatory parameters were analyzed by flow cytometry of brain immune cells and quantitative polymerase chain reaction.

**Results:**

Middle cerebral artery occlusion/reperfusion regulated *Yap*, *Taz*, and YAP/TAZ target gene expression in the brain. TAZ reporter mice confirmed stroke‐induced endothelial YAP/TAZ activation. Endothelial‐specific loss of YAP/TAZ reduced infarct volumes at 4 weeks after MCAo without impairing stroke‐induced angiogenesis, revealing an unexpected neuroprotective role for endothelial YAP/TAZ depletion. YAP/TAZ deficiency modulated cGAS−STING (cyclic GMP‐AMP synthase−stimulator of interferon genes) and Wnt (wingless‐related integration site) signaling genes in bECs and promoted myeloid cell recruitment and an anti‐inflammatory vascular environment during the subacute phase of stroke.

**Conclusions:**

Our data suggest that endothelial YAP/TAZ affects the inflammatory milieu subacutely after ischemia and thereby influences the chronic course of stroke. Modulation of YAP/TAZ activity in ECs may be a promising therapeutic target to promote neuroprotection after stroke.

**Nonstandard Abbreviations and Acronyms**
bC: brain cell
bEC: brain endothelial cell
cGAS: cyclic GMP‐AMP synthase
CTRL: control littermates (*Pdgfb‐iCreER‐eGFP* ^+/+^ *Yap* ^fl/fl^ *Taz* ^fl/fl^)
EC: endothelial cell
MCAo: middle cerebral artery occlusion
STING: stimulator of interferon genes
TAZ: WW domain containing transcription regulator 1
*Taz* ^tag^
: TAZ reporter mice (expressing a TAZ‐GFP fusion protein)
Wnt: wingless‐related integration site
YAP: yes‐associated protein 1
YT‐iKO: tamoxifen‐inducible EC‐specific *Yap*/*Taz* knockout (*Pdgfb‐iCreER‐eGFP* ^Tg/+^ *Yap* ^fl/fl^ *Taz* ^fl/fl^)

**Research Perspective**

**What Is New?**

- YAP/TAZ (yes‐associated protein 1/WW domain containing transcription regulator 1), whose relevance in the adult brain has so far been underestimated, fails to influence post‐stroke angiogenesis.
- However, loss of endothelial YAP/TAZ together with the associated altered brain‐immune response results in improved stroke outcome measures and neuroprotection at chronic, but not acute, time points.

**What Question Should Be Addressed Next?**

- Our findings expand the understanding of endothelial YAP/TAZ and may help elucidate and harness mechanisms linking YAP/TAZ and cGAS−STING (cyclic GMP‐AMP synthase−stimulator of interferon genes) signaling to improve stroke outcome.

Globally, stroke remains the second‐leading cause of death and the third‐leading cause of death and disability combined.^1^ The disease represents a major societal and economic burden.^2^ Ischemic stroke accounts for ∼62% of all strokes reported in 2019.^1^ Treatment options remain limited to thrombolysis and mechanical recanalization of the occluded vessel.^3^ Further safe and affordable treatment strategies should therefore be developed.

It is a widely accepted paradigm that acute cerebral ischemia induces brain endothelial cell (bEC) dysfunction, thereby contributing to parenchymal injury and worsened stroke outcome.^4^ On the other hand, bECs are the main drivers of stroke‐induced angiogenesis and might contribute to regenerative tissue remodeling.^5^, ^6^, ^7^, ^8^

Recently, YAP (yes‐associated protein 1) and WWTR1 (hereafter referred to as TAZ [WW domain containing transcription regulator 1]) have gained increased attention in vascular research. In ECs, YAP and TAZ are involved in proliferation, cell death, cell fate, and tissue regeneration, and their role during developmental angiogenesis has been extensively studied.^9^, ^10^, ^11^, ^12^, ^13^, ^14^ In adult organisms, endothelial YAP and TAZ are implicated in disease processes such as tumor growth, atherosclerosis, and pulmonary hypertension.^15^, ^16^, ^17^, ^18^ Although YAP and TAZ have decisive functions in ECs, their EC‐specific role in neurological diseases remains largely unknown. This is particularly true in the case of ischemic stroke, a disease whose outcome is highly dependent on EC function.

YAP and its paralog TAZ integrate mechanical, metabolic, and growth factor signals to regulate various physiological and pathophysiological processes.^19^, ^20^, ^21^, ^22^ As effectors of the canonical Hippo signaling pathway, YAP/TAZ shuttle between the cytosol and the nucleus to act as transcriptional coactivators.^20^ When the Hippo pathway is activated, YAP/TAZ are phosphorylated by LATS (large tumor suppressor kinases), resulting in their cytoplasmic retention and degradation. When the Hippo pathway is inhibited, YAP/TAZ translocate to the nucleus and bind to transcription factors, of whom TEADs (TEA domain transcription factors) are best characterized.^20^ Studies have revealed additional noncanonical signaling cascades that regulate YAP/TAZ independently of LATS.^23^ Beyond their role in transcriptional regulation, YAP and TAZ also directly modulate the innate immune response in the cytoplasm, challenging the view that YAP/TAZ function is restricted to the nucleus. Emerging evidence also suggests that YAP/TAZ interact with transcription factors and kinases in the cytoplasm in order to regulate immune signaling.^23^ For example, cytoplasmic YAP/TAZ directly interact with TBK1 (TANK‐binding kinase 1) as well as with IKK (IκB kinase ε), thereby suppressing the antiviral response. Conversely, YAP/TAZ knockdown has been shown to enhance antiviral activity.^24^

Although EC function is crucial in ischemic stroke and YAP/TAZ play pivotal roles in EC function, the impact of endothelial YAP/TAZ on chronic stroke outcome remains unexplored. The aim of the present study was to compare ischemic brain lesion sizes in control (CTRL) and induced EC‐specific *Yap*/*Taz* knockout (YT‐iKO) mice after transient middle cerebral artery occlusion (MCAo)/reperfusion. Surprisingly, loss of endothelial YAP/TAZ reduces chronic ischemic brain lesion volume even though acute magnetic resonance imaging (MRI) lesion volume is not affected. YAP/TAZ expression and activity in ECs after MCAo are highest during the subacute disease stage. Loss of YAP/TAZ promotes the reshaping of the endothelial environment^25^ by influencing local inflammation and promoting the infiltration of myeloid cells into brain tissue. Mechanistically, individual targets of the “Wnt (alternative wingless‐related integration site)‐YAP/TAZ signaling axis” and “cGAS−STING (cyclic GMP‐AMP synthase−stimulator of interferon genes) signaling axis” are highly regulated in bECs after stroke. Importantly, YAP and TAZ regulate these targets via 2 different mechanisms. First, as downstream effectors of the canonical Hippo pathway, YAP/TAZ regulate Wnt targets in the adult murine brain. However, more important, we propose that YAP/TAZ activity in the cytoplasmic matrix critically influences the cGAS‐STING signaling pathway after MCAo. Consequently, modulation of YAP/TAZ represents a promising new therapeutic target promoting anti‐inflammatory and regenerative responses after cerebral ischemia.

## METHODS

The data that support the findings of this study are available from the corresponding author upon reasonable request.

### Animals

All experimental procedures were approved by the State Office for Health and Social Affairs in Berlin and conducted following the German Animal Welfare Act and Animal Welfare Regulation Governing Experimental Animals. Mice were group housed with ad libitum access to food and water and a 12‐hour dark/light cycle. Homozygous TAZ reporter mice (*Taz* ^tag^) were described previously.^9^ In brief, a fusion tag consisting of GFP (green fluorescent protein), FLAG, and a biotin‐labeling peptide was inserted in frame upstream of the stop codon of the endogenous *Wwtr1* (*Taz*) locus. For loss of function experiments, YT‐iKO mice were studied. To this end, *Pdgfb*‐*iCreER‐eGFP* mice were crossed to *Yap* ^fl/fl^ and *Taz* ^fl/fl^.^13^, ^26^, ^27^ Littermates negative for the *Pdgfb*‐*iCreER‐eGFP* deleter allele were used as CTRLs. To trigger endothelial‐specific *Yap* and *Taz* deletion, YT‐iKO and CTRL mice received intraperitoneal tamoxifen (#T5648, dissolved in 20 mg mL^−1^ peanut oil #P2144; both from Sigma‐Aldrich) at 100 mg kg^−1^ body weight 12, 10, and 8 days before MCAo or sham surgery. Both male and female adult mice were randomly assigned to different groups in the study, and tissue samples were analyzed blindly. This research is reported following the *Animal Research: Reporting In Vivo Experiments* guidelines.^28^

### Study Design

Altogether, 4 experiments were conducted. The first experiment was performed to quantify *Yap*, *Taz*, and YAP/TAZ target gene expression at different time points after MCAo. For this experiment 31 C57BL/6N mice were used: sham (n=8), 6 hours (n=5), 24 hours (n=5), 72 hours (n=4), 7 days (n=4), and 14 days (n=5) after 30‐minute MCAo/reperfusion. No experimental animal was excluded.

The second experiment was performed to study ischemia‐induced TAZ expression. In total, 4 *Taz* ^tag^ mice were used: sham (n=1), 1 day (n=1), 3 days (n=1), 7 days (n=1) after 30‐minute MCAo/reperfusion. Again, no experimental animal was excluded.

The third experiment was conducted to study the influence of endothelial cell YAP/TAZ on stroke lesion volume at 2 days (MRI) and 28 days (NeuN [neuronal nuclei] immunohistochemistry) after MCAo. Moreover, EC numbers, vessel numbers, vessel coverage, and Iba1^+^ cell numbers were quantified in brain tissue sections at the experimental end point. This experiment included 22 mice (CTRL n=11, YT‐iKO n=11). Two mice were excluded from the study before its completion due to surgical mortality (YT‐iKO n=1) and adherence to ethical guidelines (YT‐iKO n=1).

The fourth experiment was performed to study mechanisms of YAP/TAZ‐mediated endothelial responses at the time of peak TAZ activity (3 days after MCAo). For RNA analyses, 41 animals were used: sham CTRL (n=8), sham YT‐iKO (n=8), MCAo CTRL (n=13), and MCAo YT‐iKO (n=12). Two MCAo CTRL mice (n=2) were excluded because of surgical mortality. For flow cytometry of brain cells (bCs), 19 animals were used in total. The following numbers of animals were allocated to each group: MCAo CTRL (n=9), MCAo YT‐iKO (n=10). Three mice were excluded from the study before its completion due to surgical mortality (YT‐iKO n=2) and adherence to ethical guidelines (CTRL n=1). For brain immunohistochemistry, 32 animals entered the experiment: sham CTRL (n=9), sham YT‐iKO (n=4), MCAo CTRL (n=10), and MCAo YT‐iKO (n=9). Five MCAo mice (CTRL n=2, YT‐iKO n=3) were excluded from the final analysis due to technical limitations (insufficient tissue quality for sectioning and staining). In order to collect tissue for Western blotting, 26 mice entered the experiment: sham CTRL (n=4), sham YT‐iKO (n=4), MCAo CTRL (n=8), MCAo YT‐iKO (n=10). Four mice were excluded from the study before its completion due to surgical mortality (MCAo CTRL n=1, MCAo YT‐iKO n=1) and adherence to ethical guidelines (MCAo YT‐iKO n=2). Ultimately, 3 animals per group were randomly selected for RNA sequencing‐based validation analyses. For confocal microscopy in Figures S1 through S6, 1 animal per genotype was used. For flow cytometry of white blood cells, blood was collected from the same MCAo animals used for both bC flow cytometry and brain immunohistochemistry. However, sufficient blood volume could not be obtained from 6 animals (CTRL n=3, YT‐iKO n=3).

The experimental groups, including control groups, are detailed in each figure legend. Individual mice were considered as experimental units. The number of mice per group in each experiment along with the statistical test is reported in the corresponding figure legend.

### Middle Cerebral Artery Occlusion

Cerebral ischemia was induced in adult mice by following a well‐established and standardized protocol in our laboratory, as described elsewhere.^29^, ^30^ In brief, anesthesia was induced by 1.5% isoflurane and maintained by 1.0% isoflurane in 70% N_2_O and 30% O_2_ using a vaporizer. The left MCA was occluded by introducing a 7‐0 silicone rubber‐coated monofilament (Doccol Corporation, #7019910PK5Re) into the internal carotid artery. The filament was advanced until the A1 segment of the anterior cerebral artery and thus occluded the MCA and the anterior choroidal artery. The filament was left in place for 30 minutes and then removed to allow reperfusion. Basically, the same surgical procedure was performed in sham animals, whereby the left carotid artery was exposed but not occluded. Pain management included intra‐ and postoperative treatment of the wound with 1% bupivacaine gel. The Bederson score was performed to control for neurological deficits after MCAo surgery.^31^

### Magnetic Resonance Imaging

The MRI was performed with a 7 Tesla rodent scanner Pharmascan 70/16 (Bruker BioSpin) and a 20‐mm‐1H‐RF quadrature‐volume resonator. We used a T2‐weighted 2D turbo spin‐echo sequence (imaging parameters repetition time/echo time=4200/36 ms, rare factor 8, 4 averages, 32 axial slices with a slice thickness of 0.5 mm, field of view of 2.56×2.56 cm, matrix size 256×256). Lesion volume was quantified using the Analyze v10.0 software (AnalyzeDirect). Edema‐corrected lesion volume was calculated as described previously.^32^

### Immunohistochemistry and Immunofluorescence Staining

To enable the visualization of the total cerebral vascular network at 28 days after 30‐minute MCAo/reperfusion, lectin isolated from *Lycopersicon esculentum* and conjugated to DyLight 488 (Vector Laboratories, #DL‐1174, 0.1 μg) was injected intravenously 5 minutes before mice were transcardially perfused. The mice were deeply anesthetized by intraperitoneal injection of xylazine/ketamine and transcardially perfused with physiological saline followed by 4% PFA in 0.1 M phosphate buffer, pH 7.4. Brains were dissected from the skulls and postfixed overnight. Before sectioning from a dry ice‐cooled copper block on a sliding microtome (Leica), the brains were transferred to 30% sucrose in 0.1 M phosphate buffer, pH 7.4, until they sank. Brains were cut in the coronal plane into 40 μm‐thick sections. Sections were stored at −20 °C in cryoprotectant solution (25% ethylene glycol, 25% glycerol, and 0.05 M phosphate buffer). Sections were stained using free‐floating immunohistochemistry. Primary antibodies were applied in the following concentrations: anti‐Iba1 (rabbit, Fujifilm Wako, 019‐19741) 1:500; anti‐Ki67 (rat, eBioscience, # 14‐5698‐82) 1:500; anti‐NeuN (mouse, Merck, #MAB377) 1:200; anti‐ERG (rabbit, Abcam, #ab92513) 1:100; anti‐GFP (goat, Origine, #R1091P) 1:100; isolectin B_4_ (GS‐IB_4_)—Alexa647 (isolated from *Griffonia simplicifolia*, Thermo Fisher Scientific, #I32450) 1:100; anti‐GLUT1 (rabbit, Calbiochem, #400060) 1:100; anti‐CD31 (goat, bio‐techne, #AF3628) 1:100; anti‐YAP1 (rabbit, Novus, #NB110‐58358) 1:100; anti‐Ly6G (rat, BD Biosciences, #551459) 1:100; and anti‐CD3 (rat, Bio‐RAD, #MCA1477T) 1:100. Immunohistochemistry followed the peroxidase method with anti‐rabbit IgG biotinylated secondary antibody (1:250, dianova, #711‐065‐152), ABC Elite reagent (Biozol, #VEC‐PK‐6100), and diaminobenzidine (Sigma, #D4293) as chromogen. Slides were cleared with Rotihistol (Roth, #6640.1) and coverslipped with Vitro‐Clud (Langenbrinck, #04‐0001). For immunofluorescence, anti‐rat IgG—Alexa 568 (1:400, abcam, #ab175475), anti‐rat IgG—Alexa 647 (1:400, abcam, #ab150155), anti‐rabbit IgG—Alexa 647 (1:400, Thermo Fisher Scientific, #A‐31573), anti‐goat IgG—Alexa 488 (1:200, Thermo Fisher Scientific, #A‐11055), anti‐mouse IgG—Alexa 488 (1:400, Thermo Fisher Scientific, #A‐21202), anti‐rabbit IgG—Rhod. Red‐X (1:250, dianova, #711‐295‐152), anti‐goat IgG—Rhod. Red‐X (1:250, dianova, #705‐295‐147), anti‐rabbit IgG—Alexa 568 (1:400, Thermo Fisher Scientific, #A10042), and anti‐rabbit IgG—Alexa 647 (1:400, Thermo Fisher Scientific, #A‐31573) were used. Hoechst 33342 (1 μg mL^−1^, Thermo Fisher Scientific, #H1399) was added for 10 minutes before mounting medium was added (Shandon™ Immu‐Mount™, Epredia, #9990402) or slices were mounted with antifade reagent containing DAPI (Thermo Fisher Scientific, #P36962).

### Imaging, Lesion Size and Cell Quantification

Stereo Investigator software (MBF Bioscience) was used to study NeuN lesion volume and the number of Iba1^+^ cells per area. The infarct volume was calculated by summing up the volumes of 5 40 μm‐thick NeuN‐stained coronal brain sections (2 mm apart) directly.^33^, ^34^ To count Iba1^+^ cell numbers, the ischemic lesion and the corresponding contralateral region were delineated at 100× magnification and cells were counted at 200× magnification.

For imaging *Taz* ^tag^ expression, a confocal laser scanning microscope (Leica TCS SP5) with a 20×, 0.7 NA oil objective or 63×, 1.4 NA oil objective was used. For analysis of vessel density, brain slices were imaged using a DMI8‐CS Leica confocal laser scanning microscope with a 20×, 0.75 NA dry objective. The LAS X Navigator and the mosaic merge function were used to create a 2D large scan for the entire slice. A region of interest was manually drawn in the lateral striatum of each hemisphere using the polygon selection tool in the Fiji Software.^35^ The 2 images from the selected regions of interest were cropped and moved to Imaris Software v9.7 for further analysis. The surface function was used to reconstruct the vascular structures in the image. A size filter was applied to exclude background objects <50 μm^2^. Erg‐positive cells that lie within the vascular objects were counted using the spot detection function. Then the number of vascular objects, the number of ECs, and the area covered by vessels were calculated and normalized to the area of the region of interest.

For Ki67^+^/Iba1^+^ cell counting, brain slices were imaged with a confocal laser scanning microscope (Zeiss, LSM 700) with a 20×, 0.5 NA dry objective. Two consecutive coronal slices from the center of the lesion were imaged with 4 images (z‐stacks) per hemisphere covering the lateral striatum area in the ischemic and the contralateral sides. Image analysis was conducted using an ImageJ Macro script in Fiji.^35^ The script applies a series of filters for image enhancement, followed by automated thresholding and a series of morphological filters to remove noise and background objects. Then, a distance transform watershed (3‐dimensional) was applied to separate touching objects. The function “Analyze Region 3D” from MorpholoLibJ^36^ was used to calculate the object volumes and exclude objects smaller than an empirically‐determined threshold. Boolean operations were used to detect the overlapping objects in the Iba1 and the Ki67 binary images. The overlapping objects were used as markers for a 3‐dimensional morphological reconstruction on the Ki67 image to isolate double‐positive cells (Iba1^+^/Ki67^+^). A series of morphological filters where applied again to exclude any background objects. Finally, the cell number in mm^3^ was calculated based on the image volume.

### Isolation of mRNA and Quantitative Polymerase Chain Reaction

RNA was isolated from brain tissue using TRIzol reagent (Invitrogen) or from sorted cells using the NucleoSpin RNA XS kit (Macherey‐Nagel). M‐MLV reverse transcriptase (Promega) and random hexamers (Roche) were used for reverse transcription of RNA into cDNA. For polymerase chain reaction amplification, we used gene‐specific primers (Table S1) and Light Cycler 480 SYBR Green I Master (Roche Diagnostics). Polymerase chain reaction conditions were as follows: preincubation 95 °C, 10 minutes; 95 °C, 10 seconds; primer‐specific annealing temperature, 10 seconds; 72 °C, 15 seconds (45 cycles). Crossing points of amplified products were determined using the second derivative maximum method (Light Cycler Version LCS480 1.5.0.39, Roche). Messenger RNA expression was quantified relative to either tripeptidyl peptidase 2 (*Tpp2*) in mouse specimens or receptor‐accessory protein 5 (*REEP5*) in human cells.^37^ The specificity of polymerase chain reaction products was checked using melting curve analysis. Polymerase chain reactionproducts were run on a 1.5% agarose gel to demonstrate the presence of a single amplicon of the correct size. Furthermore, negative controls (ie, reaction mix lacking either template DNA or reverse transcriptase) yielded no bands on the gel.

### Western Blot Analysis

For protein extraction, tissue or cells were harvested in RIPA lysis buffer (150 mM NaCl, 5 mM EDTA, 1% NP‐40, 1% sodium deoxycholate, 0.1% SDS, 25 mM Tris–HCl pH 7.6) containing phosphatase and protease inhibitors (PhosSTOP #04906837001, cOmplete #11836153001; both from Roche). Protein concentration was determined with the Pierce BCA Protein Assay Kit (Pierce, Thermo Fisher Scientific, #23227). Equal amounts of protein were loaded on 4% to 20% precast polyacrylamide gels (Bio‐Rad, #4561096) and blotted onto PVDF membranes (Immobilon, Millipore, Merck, #IPFL 07810) for 130 minutes at 25 V. Blots were probed with primary and secondary antibodies and developed using near‐infrared fluorescence detection with Odyssey CLx Imaging System (LI‐COR). Equal loading of protein was confirmed by blotting against GAPDH. Primary and secondary antibodies were applied in the following dilutions: CXCL10 (Bio‐Techne, #AF‐466) 1:200; GAPDH (Sigma, #MAB374) 1:2000; STING (Cell Signaling, #50474) 1:1000; TBK1 (Cell Signaling, #3504) 1:1000; P‐TBK1 (Cell Signaling, #5483) 1:1000; IRDye 800 (LI‐COR) 1:15 000, and IRDye 680 (LI‐COR) 1:15 000.

### Human Umbilical Vein Endothelial Cell Culture

Human umbilical vein ECs from pooled donors (PromoCell) were cultured in EGM‐2 Endothelial Cell Growth Medium‐2 BulletKit without antibiotics (Lonza, #CC‐3162) and used until passage 7. For knockdown experiments, human umbilical vein ECs were transfected with SMARTpool: siGENOME siRNAs purchased from Dharmacon (Yap #M‐012200‐00‐0005, Taz #M‐016083‐00‐0005, CTRL nontargeting siRNA Pool 1 #D001206‐13‐05). Subconfluent (70%–80%) human umbilical vein ECs were transfected with 25 nM siRNA using Dharmafect 1 transfection reagent following the protocol from the manufacturer; transfection media was removed after 24 hours. To activate STING signaling, cells were treated with the STING agonist 2′,3′‐cGAMP (5 μg mL^−1^, Tocris, #5945) or vehicle (H_2_O, 1:1000).

### Cell Isolation, Blood Collection and Flow Cytometry

In order to isolate and sort bCs, YT‐iKO and CTRL mice were deeply anesthetized by intraperitoneal injection of xylazine/ketamine followed by transcardial perfusion with PBS. Brains were quickly removed, and sham tissue or the ipsilateral ischemic MCA territory, along with the corresponding area in the contralateral hemisphere, was carefully dissected and placed in cold PBS (4 °C). Tissue was dissociated with neural tissue dissociation kit (Miltenyi Biotec, #130‐092‐628). bEC isolation has been described previously.^38^ In brief, myelin debris was removed by myelin removal beads (Miltenyi Biotec, #130‐096‐733). CD31^+^ enriched bECs were isolated by magnetic associated cell sort using CD31 microbeads (Miltenyi Biotec, #130‐097‐418). Unlabeled CD31^−^ bCs that run through a MS column (Miltenyi Biotec, #130‐042‐201) were collected for quantitative real‐time polymerase chain reaction. Eluted CD31^+^ bECs were further purified by CD45^−^, DAPI^−^, CD31^+^ flow sorting using a FACSAria II (BD Biosciences). To isolate brain immune cells for flow cytometry analysis, myelin debris was removed after tissue dissociation by Percoll density gradient centrifugation. Microglia and central nervous system‐resident leukocytes were collected from the 70% to 30% interphase, washed and processed by immunostaining. Blood was collected for flow cytometry analysis in K_3_EDTA‐tubes (Sarstedt, #20.1341.100). Within 30 minutes, blood was transferred into polystyrene tubes (Falcon, #352235) and stained with respective antibodies. Red blood cells were lysed with BD Pharm Lyse buffer (BD Biosciences, #555899). Washed brain and blood samples were resuspended in FACS buffer (PBS+0.5% BSA) and run on the FACSCanto II flow cytometer (BD Biosciences). Data were analyzed with the FlowJo software v10 (BD Biosciences). The following dead cell stains and antibodies (all from BioLegend) were used: SYTOX green (Thermo Fisher Scientific, #S7020, 1:1000), DAPI (Miltenyi Biotec, #130‐111‐570, 1:100), CD45‐APC (#103112, 1:100), CD31‐PE/Cy7 (#102523, 1:100), CD45‐PE (#103106, 1:400), CD11b‐APC (#101212, 1:100), CD172a‐APC/Fire (#144030, 1:100), CD8‐PE/Cy7 (#100722, 1:100), CD3ε‐PerCP/Cy5.5 (#152311, 1:100), CD19‐BV421 (#115537, 1:100), CD4‐BV510 (#100553, 1:100), Ly6C‐PE/Cy7 (#128018, 1:800), CD115‐BV421 (#135513, 1:50), Ly6G‐BV510 (#127633, 1:100), and CD16/32‐TruStain FcX (#101319, 1:50).

### RNA Sequencing

RNA was isolated from freshly FACsorted bEC using NucleoSpin RNA XS columns (Machery‐Nagel) without carrier RNA and eluted in ultrapure water. RNA quality has been evaluated by High Sensitivity RNA ScreenTape analysis (Agilent Technologies). RNA libraries were prepared using SMART‐Seq Ultra Low Input RNA kit (TaKaRa). RNAseq was done on an Illumina NovaSeq6000 platform in a 101+8+8+101 nt paired‐end mode (Illumina). Sequences were mapped to mouse genome, v. GRCm38 p5 with GENCODE annotation v. M12 using the program STAR, version 2.7.8a.

Differentially expressed genes (DEG) were identified using DEseq2 R package, v. 1.38. DEG for each contrast were defined by *P*adj values <0.1. For gene set enrichment analysis we used the CERNO algorithm as implemented in the tmod R package v. 0.50.7.^39^, ^40^ Gene ontology profiling of gene lists was done using ShinyGO 0.77 online tool.^41^ RNA sequencing data have been deposited at Gene Expression Omnibus with the data set identifier GSE265949.

### Statistical Analysis

Statistical analysis was performed using GraphPad Prism 10.2.2. Normality testing was done by Shapiro–Wilk test. Data are presented as means±SD or as medians for parametric and nonparametric statistical tests, respectively. Groups were compared by statistical tests as indicated in the figure legends. Differences were considered statistically significant with *P*<0.05.

## RESULTS

### YAP/TAZ and Their Downstream Signaling Targets Show Distinct Spatio‐Temporal Expression Patterns in the Brain After Ischemia

**Figure 1:**
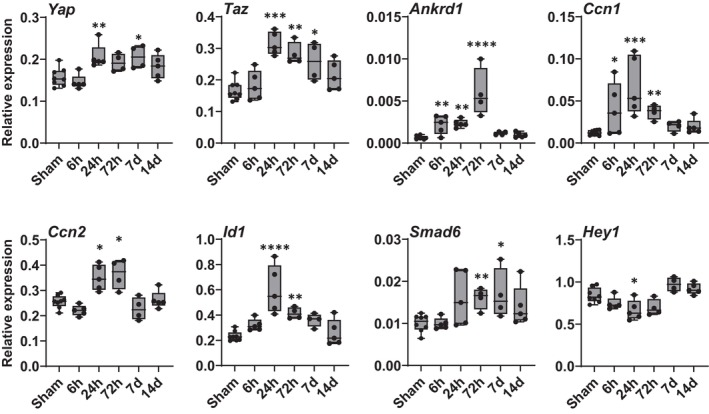
Ischemia‐induced YAP and TAZ signaling pathway genes. Differential gene expression of *Yap* and *Taz* and YAP/TAZ target genes (*Ankrd1*, *Ccn1*, *Ccn2*, *Id1*, *Smad6*, *Hey1*) in ipsilateral stroke tissue at different time points (6 h, 24 h, 72 h, 7 d, 14 d) after 30‐minute MCAo or in corresponding tissue after sham surgery. *Y* axis shows mRNA expression relative to housekeeping gene *Tpp2*, as quantified by quantitative reverse transcription polymerase chain reaction. Nonparametric 1‐way ANOVA (Kruskal–Wallis test) with uncorrected Dunn’s post hoc test, n=4 to 8 mice per group, *P<0.05, **P<0.01, ***P<0.001, ****P<0.0001 sham vs MCAo/reperfusion time point. Box‐and‐whiskers plots (box extends from the 25th to 75th percentiles), with median and minimum to maximum value whiskers. All data points are superimposed on the graph. *Ankrd1* indicates ankyrin repeat domain 1; *Ccn1*, cellular communication network factor 1; *Ccn2*, cellular communication network factor 2; *Hey1*, hairy/enhancer‐of‐split related with YRPW motif 1; *Id1*, inhibitor of DNA binding 1, HLH protein; *Smad6*, SMAD family member 6; *Taz*, WW domain containing transcription regulator 1; *Tpp2*, tripeptidyl peptidase 2; and *Yap*, yes‐associated protein 1.

**Figure 2:**
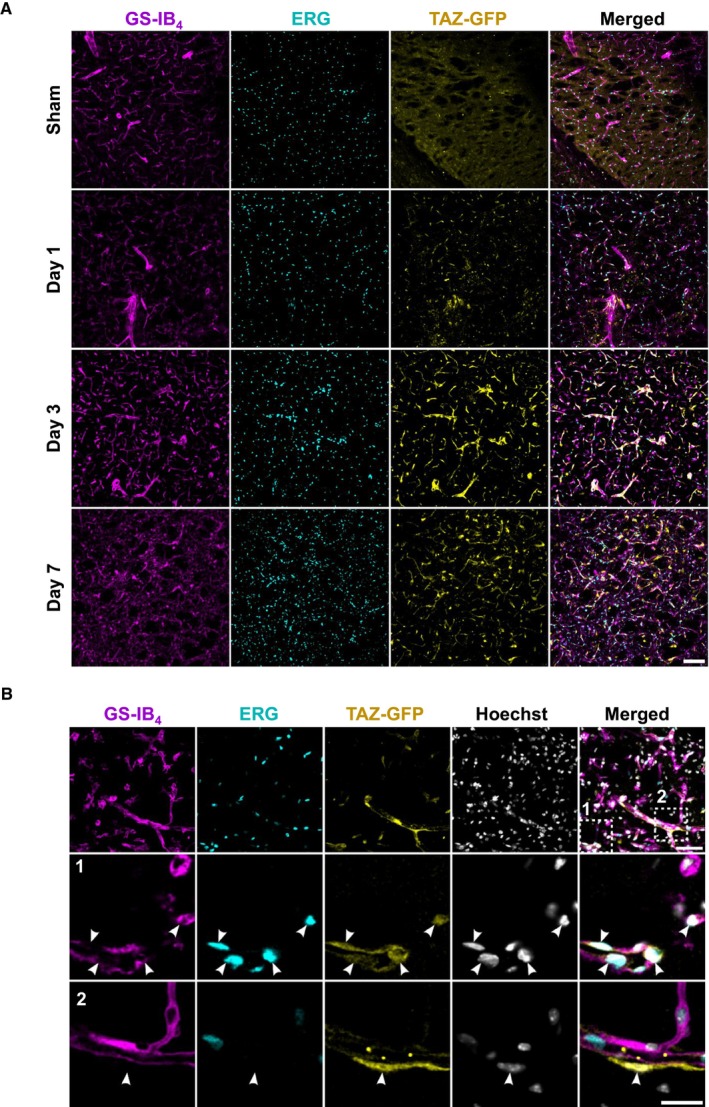
Ischemia‐induced TAZ expression. **A**, TAZ‐GFP reporter expression in sham and MCAo *Taz* ^tag^ mice at 1, 3, or 7 d of reperfusion. Representative images as MIPs taken from the ischemic region in the lateral striatum of MCAo mice and the corresponding counterpart in the sham animals. Images show an upregulation of the TAZ‐GFP reporter (yellow) after stroke, which peaked at 3 d post injury. GS‐IB_4_ (magenta) and ERG (turquoise) as vascular and endothelial counterstains. Scale bar: 100 μm. **B**, High magnification images showing the nuclear translocation of the TAZ‐GFP reporter in both ERG^+^ endothelial cells (inset 1, white arrowheads) and ERG^−^ nonendothelial cells (inset 2, white arrowheads) 3 d after reperfusion. Hoechst as nuclear counterstain. Upper row shows a MIP, and the magnified images show a single z‐plane. Scale bars: 50 μm in the upper row and 20 μm in the magnified images. ERG indicates ETS transcription factor; GFP, green fluorescent protein; GS‐IB_4_, isolectin B_4_ isolated from *Griffonia simplicifolia*; MCAo, middle cerebral artery occlusion; MIP, maximum intensity projection; TAZ, WW domain containing transcription regulator 1; and *Taz* ^tag^, TAZ reporter mice.

To understand whether YAP/TAZ signaling is altered by MCAo, we performed mRNA quantification of *Yap*, *Taz*, and several downstream YAP/TAZ‐TEAD targets such as *Ankrd1* (ankyrin repeat domain 1) , *Ccn1* (cellular communication network factor 1), *Ccn2* (cellular communication network factor 2), *Id1* (inhibitor of DNA binding 1), *Smad6* (SMAD family member 6), and *Hey1* (hairy/enhancer‐of‐split related with YRPW motif 1).^13^ The data showed differential expression patterns in ischemic brain tissue during the subacute disease phase (Figure 1). In comparison to sham‐operated mice, MCAo mice showed significantly increased expression of *Yap* (24 hours and 7 days), *Taz* (24 hours, 72 hours, 7 days), *Ankrd1* and *Ccn1* (6 hours, 24 hours, 7 days), *Id1* and *Ccn2* (24 hours, 72 hours) along with *Smad6* (72 hours, 7 days) in brain tissue (Figure 1). In contrast, *Hey1* expression was significantly reduced 24 hours after MCAo (Figure 1). We then used a TAZ reporter mouse^9^ to study the expression and subcellular localization of TAZ in the brain. In comparison to mice that had undergone sham surgery, TAZ expression in the ischemic lateral striatum demonstrated a slight increase as early as day 1 following MCAo. By day 3, a clear and abundant signal was observed, which subsequently decreased again on day 7 (Figure 2A). Colabeling with ERG (ETS transcription factor ERG), which stains endothelial nuclei, and GS‐IB_4_, which stains the vasculature, confirmed that the TAZ signal derives from the brain vasculature (Figure 2A and 2B). Within the vasculature, TAZ co‐localizes with the nucleus of both ECs and non‐ECs (Figure 2B).

### Loss of Endothelial YAP/TAZ Reduces the Size of Chronic Ischemic Brain Lesions

**Figure 3:**
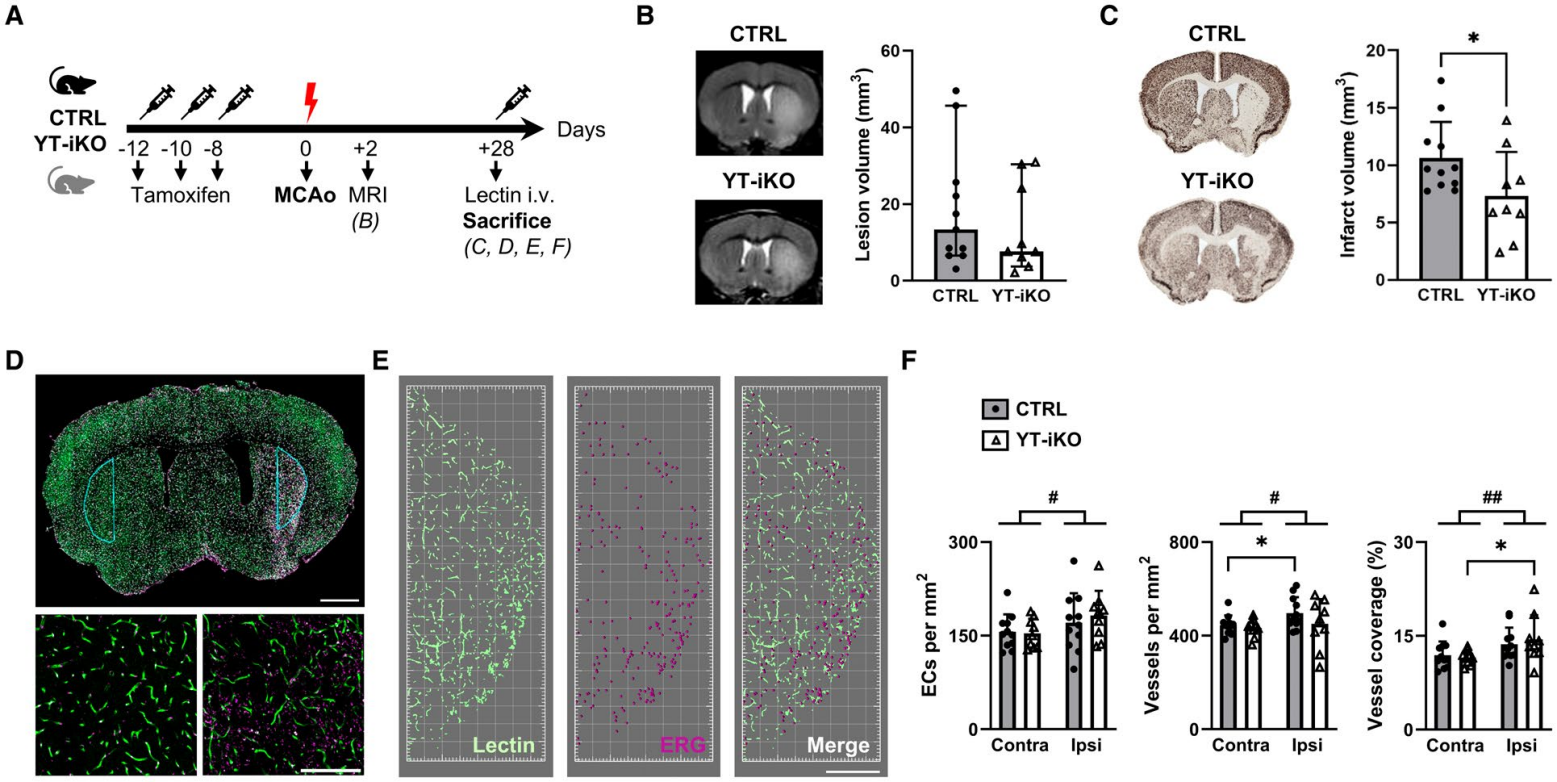
Loss of YAP/TAZ in endothelial cells reduces chronic stroke volume. **A**, Experimental design. Mice received tamoxifen 12, 10 and 8 d before MCAo followed by reperfusion. Two days after MCAo/reperfusion, acute lesion volume was measured using T2‐weighted MRI. Before being euthanized on day 28 after MCAo, mice received lectin to stain brain vasculature. **B**, Infarct volumetry based on MRI measurements in CTRL (n=11) and YT‐iKO mice (n=9) 2 d after MCAo. Median with 95% CI, Mann–Whitney test, *P*=0.4561. **C**, Infarct volumetry based on NeuN immunohistochemistry in CTRL (n=11) and YT‐iKO mice (n=9) 28 d after MCAo. Mean±SD, unpaired *t* test (2 tailed), *P<0.05. **D**, **Upper** part, representative image of a large scan of a brain slice. The selected ROIs for vessel density quantification (**E**, **F**) in both ipsi‐ and contralateral hemispheres appear in blue. Scale bar: 1 mm. **Lower** part, magnified images for the lateral striatum in the contralateral (left) and ischemic (right) hemispheres. Scale bar: 200 μm. Lectin‐perfused vessels labeled in green and endothelial cell nuclei labeled in magenta with ERG immunohistochemistry. **E**, Rendering of the vasculature in green and the overlapping endothelial cell nuclei in magenta in the stroke ROI in the lateral striatum segmented with Imaris Software. Vessels appear in green and the overlapping endothelial cell nuclei in magenta. Scale bar: 300 μm. **F**, Quantification of the number of vessel‐associated ECs per mm^2^, the number of vessels per mm^2^, and the percentage coverage of vessels normalized to the area of ROI in CTRL (n=11) and YT‐iKO mice (n=9) 28 d after MCAo. Mean±SD, 2‐way repeated measures ANOVA (^#^ *P*<0.05, ^##^ *P*<0.01 contra vs ipsi) followed by uncorrected Fisher’s least significant difference post hoc test: *P<0.05. CTRL indicates control littermates; EC, endothelial cell; MCAo, middle cerebral artery occlusion; MRI, magnetic resonance imaging; NeuN, neuronal nuclei; ROI, region of interest; TAZ, WW domain containing transcription regulator 1; YAP, yes‐associated protein 1; and YT‐iKO, tamoxifen‐inducible EC‐specific *Yap*/*Taz* knockout.

To better understand the functional role of YAP/TAZ in the endothelium, we next made use of YT‐iKO mice and littermate wildtype CTRLs.^13^ Mice were injected with tamoxifen followed by the MCAo/reperfusion procedure before brain tissue was analyzed (Figure 3A). Endothelium‐specific transgene expression and successful recombination were confirmed in YT‐iKO mice (Figures S1A, S1B, and S2A). Furthermore, immunohistochemical analysis of CTRL mice revealed that the transient TAZ expression, as shown in Figure 2A, was accompanied by distinct YAP expression in the vasculature of ischemic brain tissue 3 days after MCAo. However, this expression was not observed in contralateral vessels or in vessels 28 days after MCAo (Figures S2A and S3). Noninvasive MRI measurements 2 days after MCAo showed no statistically significant difference in edema‐corrected lesion sizes between CTRL and YT‐iKO mice (Figure 3B). However, the ischemic brain lesion volumes quantified by NeuN staining at 28 days after MCAo revealed a neuroprotective phenotype of YT‐iKO mice with a significant reduction in mean NeuN^−^ lesion size compared with CTRL mice (Figure 3C). This indicates that the lesion refers to the region showing a loss of NeuN‐stained neuronal nuclei, implying that these lesions are NeuN‐negative. Because YAP/TAZ depletion in ECs is known to impair neonatal retinal angiogenesis,^13^ we next studied whether loss of YAP/TAZ also leads to differences in vessel morphology during stroke‐induced angiogenesis. Therefore, we analyzed images of lectin‐perfused vessels in immunostained brain sections from CTRL and YT‐iKO mice 28 days after stroke (Figure 3D through 3F). MCAo surgery had a significant effect on EC number, vessel number, and vessel coverage in CTRL and YT‐iKO mice. However, YAP/TAZ deficiency had no influence on EC number, vessel number, and vessel coverage in the ipsilateral striatum (Figure 3F). Additional histological analysis of CD31 and GLUT1‐immunostained brain sections revealed no signs of abnormalities in the vascular network of YT‐iKO mice (Figures S1C and S2B). These results strongly suggest that endothelial YAP/TAZ deficiency in adult mice may promote post‐stroke neuroprotection via mechanisms independent of angiogenesis.

### Loss of YAP/TAZ Influences the Transcriptome of ECs and Modulates Inflammatory Gene Expression of the Brain Environment

**Figure 4:**
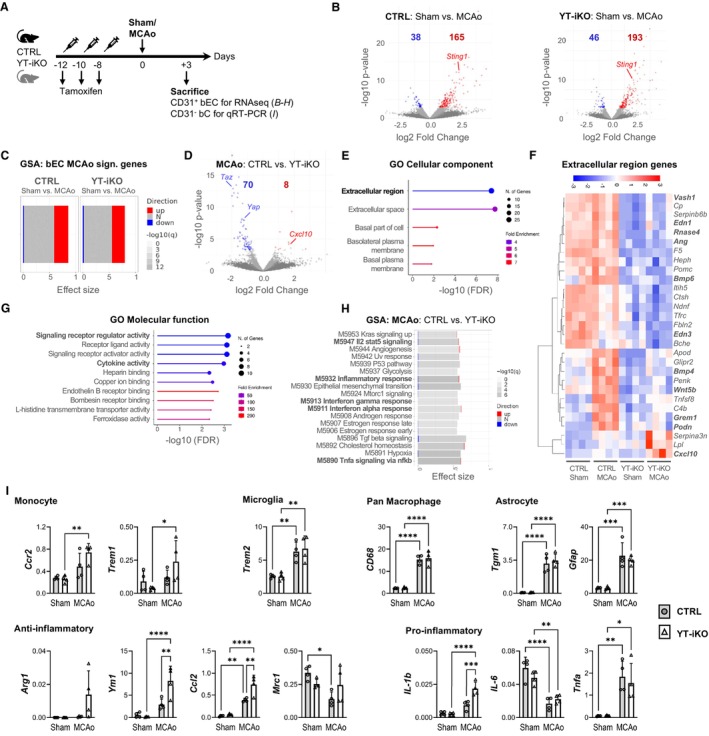
RNA analysis of CD31^+^ bECs and CD31^−^ bCs 3 days after 30‐minute MCAo/reperfusion. **A**, Experimental design. Mice received tamoxifen 12, 10, and 8 d before MCAo or sham surgery. Three days after surgery, CD31^+^ bECs and CD31^−^ bCs were isolated. **B**, Volcano plot depicting DEGs in CTRL bECs (left) and YT‐iKO bECs (right) after MCAo. Blue dots indicate downregulated transcripts after experimental stroke (*P*adj<0.1). Red dots indicate upregulated transcripts after experimental stroke (*P*adj<0.1). **C**, GSA for the bEC‐MCAo signature gene set.^38^ Bar length indicates effect size (area under curve); column corresponds to contrast, and intensity of the color corresponds to the FDR; red and blue fragments indicate fraction of DEGs within the contrast (*P*adj<0.1). **D**, Volcano plot depicting DEGs between YT‐iKO vs CTRL bECs from MCAo mice. **E**, GO overrepresentation analyses (cellular component) of 78 DEGs from the contrast YT‐iKO vs CTRL bECs isolated from MCAo mice. **F**, Heatmap of 29 DEGs associated with the GO term “extracellular region” from the contrast YT‐iKO vs CTRL bECs isolated from MCAo mice. **G**, GO overrepresentation analyses (molecular function) of 78 DEGs from the contrast YT‐iKO vs CTRL bECs isolated from MCAo mice. **H**, GSA for the contrast YT‐iKO vs CTRL bECs from MCAo mice. Row corresponds to enriched molecular signature database term (hallmark), bar length indicates effect size (area under curve), intensity of the color corresponds to the FDR; red and blue fragments indicate fraction of DEGs within the contrast (*P*adj<0.1). **I**, Gene expression analysis of cell markers and inflammatory markers in CD31^−^ nonendothelial brain cells. *Y* axis shows mRNA expression relative to housekeeping gene *Tpp2*, as quantified by qRT‐PCR. Please note the significant increase of anti‐inflammatory and monocyte markers in YT‐iKO mice after MCAo. Mean±SD, n=4 independent donors, 2‐way ANOVA followed by Tukey’s post hoc test, *P<0.05, **P<0.01, ***P<0.001, *P*<0.00001. *Arg1* indicates arginase, liver; bC, brain cell; bEC, brain endothelial cell; *Ccl2*, C‐C motif chemokine ligand 2; *Ccr2*, C‐C motif chemokine receptor 2; *CD68*, macrosialin; *Cxcl10*, C‐X‐C motif chemokine ligand 10; CTRL, control littermates; DEG, differentially expressed gene; FDR, false discovery rate; *Gfap*, glial fibrillary acidic protein; GO, gene ontology; GSA, gene set enrichment analysis; *IL‐1b*, interleukin 1 beta; *IL‐6*, interleukin 6; MCAo, middle cerebral artery occlusion; *Mrc1*, mannose receptor C‐type 1; qRT‐PCR, quantitative reverse transcription polymerase chain reaction; *Sting1*, stimulator of interferon response cGAMP interactor 1; *Tgm1*, transglutaminase 1; *Tnfa*, tumor necrosis factor; *Trem1*, triggering receptor expressed on myeloid cells 1; *Trem2*, triggering receptor expressed on myeloid cells 2; *Ym1*, chitinase‐like 3; and YT‐iKO, tamoxifen‐inducible EC‐specific *Yap*/*Taz* knockout.

To gain further mechanistic insights into the neuroprotective phenotype observed in YT‐iKO mice, we performed bulk RNA sequencing analysis of freshly isolated bECs from YT‐iKO and littermate wildtype CTRLs for sham and MCAo groups in quadruplicate, respectively (Figure 4A; Figure S4A and S4B). To obtain sufficient cell material for bEC isolation (ie, MACsort, FACsort) and subsequent RNA sequencing, brain tissue of 2 to 4 mice was pooled for each replicate (Figure S4A and S4B). Differential gene expression analysis revealed downregulation of *Yap*, *Taz* and YAP/TAZ target genes in sham‐operated YT‐iKO bECs, indicating successful Cre‐loxP recombination in YT‐iKO mice after tamoxifen injections (Figure S4C). To examine stroke‐induced changes, bECs from MCAo mice were compared with those from sham‐operated mice of the same genotype. A total of 165 upregulated and 38 downregulated transcripts were identified in CTRL mice, and 193 upregulated and 46 downregulated transcripts were identified in YT‐iKO mice (Figure 4B; Table S2). Gene set enrichment analysis for a custom bEC‐MCAo signature gene set previously published by our group,^38^ showed a high similarity with bECs from CTRL (effect size 0.86, *P*adj<2e‐16) and YT‐iKO mice (effect size 0.8, *P*adj<2e‐16) after MCAo (Figure 4C).^38^ An additional gene set enrichment analysis revealed that stroke induced endothelial and stroke‐specific modules, such as M5944 (angiogenesis), M5930 (epithelial mesenchymal transition), M5897 (IL6 Jak Stat3 signaling), M5890 (TNFa [tumor necrosis factor alpha] signaling via NF‐κB [nuclear factor kappa B]), and M5891 (hypoxia) in both CTRL and YT‐iKO bECs after MCAo (Figure S4D). To determine the relevant consequences of YAP and TAZ loss in MCAo treated bECs, we compared bECs isolated from CTRL and YT‐iKO mice after MCAo. In total, 78 differentially expressed transcripts (*P*adj<0.1; 70 downregulated, 8 upregulated) were found (Figure 4D; Table S2). To obtain an overview of the functional classification of the 78 DEGs, gene ontology analyses were performed (Figure 4E). Analyses for the gene ontology cellular component revealed an overrepresentation of the term “extracellular region”, including 29 genes out of 78 DEGs. The expression of these 29 genes across all experimental groups was visualized using a heatmap (Figure 4F). It appears that the majority of the extracellular region genes had reduced expression levels after YAP/TAZ loss, irrespective of brain ischemia (eg, *Vash1*, *Edn1*, *Edn3*, *Rnase4*, *Ang*, *Bmp6*). Remarkably, several MCAo‐regulated genes in CTRL bECs such as *Bmp4*, *Wnt5b*, *Grem1*, and *Podn* were not induced in YT‐iKO bECs. In contrast, the chemokine *Cxcl10* was exclusively upregulated in YT‐iKO bECs 3 days after MCAo (Figure 4D and 4F; Figure S4F). Interestingly, *Sting1*, which encodes the upstream activator of *Cxcl10*, was upregulated after MCAo in both YT‐iKO and CTRL bECs (Figure 4B and 4C; Figure S4F). Further gene ontology analysis using the molecular function database for 78 DEGs between CTRL bECs and YT‐iKO bECs after stroke revealed that overrepresented terms were associated with paracrine functions such as signaling receptor regulator activity and cytokine activity (Figure 4G). In line with these findings, additional gene set enrichment analysis of MCAo CTRL versus MCAo YT‐iKO bECs identified 5 significantly enriched gene sets related to inflammatory pathways (M5947 IL2‐Stat5 Signaling, M5932 Inflammatory Response, M5913 Interferon Gamma Response, M5911 Interferon Alpha Response, M5890 Tnfa Signaling via NF‐κB) (Figure 4H). A common characteristic of the aforementioned inflammatory pathways is an overlapping gene signature with *Cxcl10* as a candidate molecule differentially regulated in YT‐iKO bECs after MCAo (Figure S4E). Taken together, the transcriptomic data indicate that loss of YAP and TAZ in bECs causes changes that affect the extracellular compartment and signaling receptor/cytokine activity, likely by altered paracrine signaling. Therefore, we hypothesize that the vascular endothelial niche after stroke is altered by the loss of endothelial YAP/TAZ. Consequently, endothelial‐depleted bCs of the CD31^−^ MACsort fraction were analyzed from the same mice that were used for EC‐specific RNA sequencing (CD31^+^ MACsort fraction) to test whether the bEC environment differs between CTRL and YT‐iKO after stroke (Figure S4A and S4B). Quantitative real‐time polymerase chain reaction against several cellular and inflammatory markers was performed (Figure 4I). Microglia marker *Trem2*, astrocyte markers *Gfap* and *Tgm1*, panmacrophage marker *CD68*, and proinflammatory markers *Tnfa* and *IL‐6* were significantly upregulated after MCAo in CD31^−^ bCs of both CTRL and YT‐iKO mice. However, no influence of YAP/TAZ loss was detectable for any of the markers mentioned. In contrast, monocyte‐specific mRNA levels such as *Ccr2* and *Trem1* were significantly increased exclusively in YT‐iKO CD31^−^ bCs after MCAo. Consistent with the increase of monocytic genes, anti‐inflammatory markers such as *Ym1* and *Ccl2* were significantly upregulated in YT‐iKO CD31^−^ bCs compared with CTRL CD31^−^ bCs after MCAo. Interestingly, downregulation of anti‐inflammatory *Mrc1* expression was attenuated in YT‐iKO CD31^−^ bCs after MCAo. Furthermore, *IL‐1b*, a proinflammatory marker known to increase the opening of the blood–brain barrier (BBB),^42^, ^43^, ^44^, ^45^ was significantly increased in YT‐iKO CD31^−^ bCs. In conclusion, our study demonstrates that loss of YAP and TAZ in bECs modulates the inflammatory brain environment after stroke. A statistically significant increase in monocytic markers and an anti‐inflammatory gene expression profile is evident in the vascular endothelial niche of YT‐iKO mice following MCAo.

### Endothelial YAP/TAZ Affects cGAS‐STING Pathway Controlled *Cxcl10* Expression

**Figure 5:**
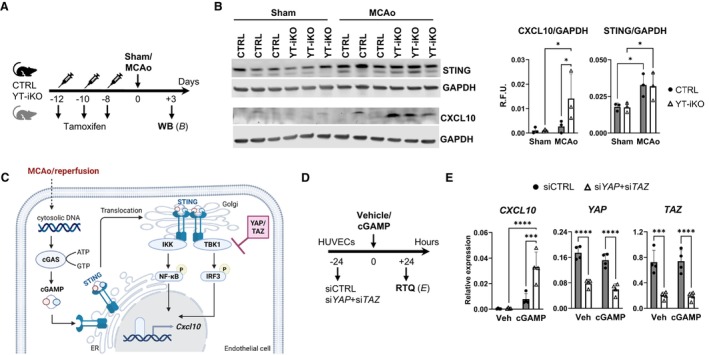
STING pathway activation after stroke and YAP/TAZ‐mediated STING pathway interference in HUVECs. **A**, Experimental design. Brain tissue from CTRL and YT‐iKO mice was collected for WB 3 d after sham or MCAo surgery. **B**, WB demonstrates increased STING expression after MCAO in CTRL and YT‐iKO brain tissue, whereas CXCL10 expression is significantly increased in ischemic YT‐iKO after stroke. Mean±SD, n=3 independent donors, 2‐way ANOVA followed by uncorrected Fisher’s least significant difference test, *P<0.05. **C**, Visualization of proposed YAP/TAZ effects on stimulator of IFN (interferon) genes (STING) pathway in endothelial cells after stroke. Ischemic brain injury/reperfusion induces an increase in double‐stranded DNA levels in the cytosol.^46^ Cytosolic DNA acts as a damage‐associated molecular pattern and is recognized by cGAS.^48^ Upon DNA binding, cGAS catalyzes the synthesis of cGAMP, which in turn activates STING and its translocation from the ER to the Golgi apparatus.^48^ There, it recruits and activates TBK1 and IKK.^47^ The kinase activities of IKK and TBK1 act downstream on transcriptional regulators NF‐κB and IRF3.^47^ In endothelial cells, STING‐mediated transcriptional activation after MCAO is negatively regulated by YAP/TAZ. Accordingly, depletion of YAP/TAZ promotes *Cxcl10* expression. Created in BioRender. Göttert, R. (2025) https://BioRender.com/gamdctf. **D**, Experimental design. HUVECs were treated after siRNA‐induced *YAP*/*TAZ* knockdown for 24 h with vehicle or cGAMP. **E**, Gene expression of *CXCL10*, *YAP*, and *TAZ* in CTRL and *YAP*/*TAZ* knockdown cells. Expression normalized to housekeeping gene *REEP5*. N=4, 2‐way ANOVA followed by uncorrected Fisher’s least significant difference test, ***P<0.001, ****P<0.00001. cGAMP indicates cyclic guanosine monophosphate–adenosine monophosphate; cGAS, cyclic GMP‐AMP synthase; CXCL10, C‐X‐C motif chemokine ligand 10; HUVEC, human umbilical vein endothelial cell; IKK, IκB kinase; IRF3, interferon regulatory factor 3; MCAo, middle cerebral artery occlusion; NF‐κB, nuclear factor‐κB; *REEP5*, receptor accessory protein 5; R.F.U., relative fluorescence units; RTQ, quantitative polymerase chain reaction; STING1, stimulator of interferon response cGAMP interactor 1; TAZ, WW domain containing transcription regulator 1; TBK1, TANK‐binding kinase 1; WB, Western blotting; YAP, yes‐associated protein 1; and YT‐iKO, tamoxifen‐inducible EC‐specific *Yap*/*Taz* knockout.

To further investigate the intriguing *Sting1* and *Cxcl10* expression patterns observed in CTRL and YT‐iKO bECs, we sought to determine whether STING and CXCL10 protein levels are significantly altered in ischemic brain tissue. Therefore, ipsilateral ischemic cerebral tissue and corresponding tissue from sham‐operated CTRL and YT‐iKO mice were analyzed by Western blotting (Figure 5A and 5B). STING protein levels were significantly elevated in CTRL and YT‐iKO brains 3 days after MCAo, whereas CXCL10 expression was increased only in YT‐iKO tissue. These findings highlight the contribution of bECs to overall STING and CXCL10 expression following MCAo (Figure 5B). Available literature suggests that YAP/TAZ functions as a negative regulator of the cGAS‐STING pathway, a mediator of inflammation in the context of brain ischemia (Figure 5C).^24^, ^46^, ^47^, ^48^ To further validate this hypothesis, an in vitro model was established in which *YAP*/*TAZ* were knocked down in human umbilical vein ECs, followed by treatment with the STING agonist cGAMP (Figure 5D). This knockdown led to a significant increase in STING‐mediated *CXCL10* expression (Figure 5E). Together, these results demonstrate that YAP/TAZ deficiency disrupts a regulatory component of the cGAS‐STING signaling pathway in endothelial cells.

### Loss of YAP/TAZ Increases the Infiltration of the Brain With Myeloid Cells and Attenuates Microglia Proliferation

**Figure 6:**
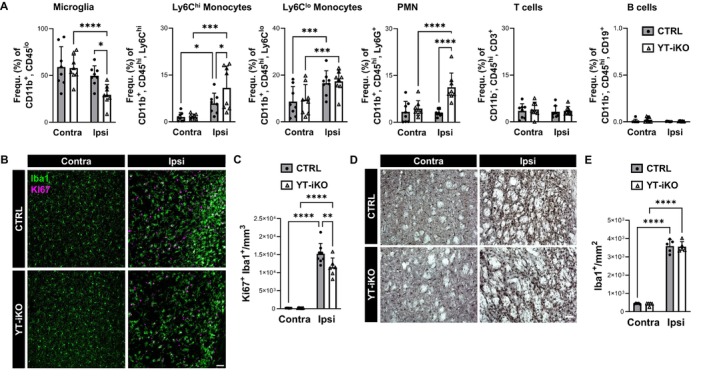
Distinct differences in myeloid cell infiltration and microglia proliferation during subacute stroke. **A**, FACS analysis of selected myeloid and lymphocyte cell populations in brain tissue 3 d after stroke onset. All cell frequencies (%) were calculated from CD45^+^ living single cells. N=8 per group. **B**, **C**, Representative confocal images (**B**) and quantification (**C**) of Ki67^+^ (magenta) and Iba1^+^ (green) double immunostainings of ipsilateral and contralateral striatum of CTRL (n=8) and YT‐iKO (n=6) mice 3 d after MCAo. **D**, **E**, Representative brightfield images of Iba1‐DAB immunohistochemistry (**D**) and Iba1^+^ cell counting (**E**) in the ipsilateral and contralateral striatum 28 d after stroke in YT‐iKO (n=5) and CTRL (n=5) mice. **A**, **C**, **E**, Two‐way repeated measures ANOVA followed by uncorrected Fisher’s least significant difference test; mean±SD; *P<0.05, **P<0.01, ***P<0.001, ****P<0.0001. **B**, **D**, Scale bar: 50 μm. CTRL indicates control littermates; FACS, fluorescence‐activated cell sorting; MCAo, middle cerebral artery occlusion; and YT‐iKO, tamoxifen‐inducible EC‐specific *Yap*/*Taz* knockout.

Given that YT‐iKO mice show elevated expression of anti‐inflammatory and monocyte markers after MCAo, we further investigated the impact of endothelial YAP/TAZ deficiency on the immune cell composition in both the brain and blood. To this end, we quantified different immune cell populations of CTRL and YT‐iKO mice by flow cytometry (Figure 6A; Figure S5A through S5E). Quantification of brain immune cells revealed that the frequency of CD11b^+^, CD45^lo^ microglia was significantly reduced in the ischemic hemisphere after MCAo in YT‐iKO mice compared with CTRL littermates (Figure 6A; Figure S5E). In contrast, classical monocytes (CD11b^+^, CD45^hi^, Ly6G^−^, Ly6C^hi^) showed higher frequencies in the ischemic hemisphere after MCAo in YT‐iKO compared with CTRL littermates (Figure 6A). Nonclassical monocytes (CD11b^+^, CD45^hi^, Ly6G^−^, Ly6C^lo^) showed higher frequencies in the ipsilateral hemisphere but no differences between YT‐iKO and CTRL mice (Figure 6A). Polymorphonuclear neutrophil (CD11b^+^, CD45^hi^, Ly6C^mid^, Ly6G^+^) frequencies were significantly increased in the ischemic striatum of YT‐iKO mice (Figure 6A). Since the neurovascular unit acts as a barrier for polymorphonuclear neutrophils,^49^ we performed additional immunohistochemistry to visualize ischemic tissue and associated polymorphonuclear neutrophil accumulation using the marker Ly6G in YT‐iKO and CTRL mice (Figure S6A). Ly6G^+^ cells were restricted to vascular structures in ischemic brain tissue, indicating that they did not invade the brain parenchyma. T cells (CD45^hi^, CD11b^−^, CD19^−^, CD3^+^) and B cells (CD45^hi^, CD11b^−^, CD3^−^, CD19^+^) were detected at low frequencies in brain tissue and were not influenced by MCAo or loss of YAP and TAZ (Figure 6A). Immunofluorescence confocal microscopy visualized very rare T cell accumulation of CD3^+^ cells in the ischemic tissue in YT‐iKO and CTRL mice (Figure S6B). In the blood, classical monocyte, T cell and B cell frequencies were not influenced by loss of YAP/TAZ after MCAo, but polymorphonuclear neutrophils and nonclassical monocytes were significantly altered (Figure S5A through S5D). In order to further investigate the unexpected finding that microglia frequencies were reduced in the ipsilateral striatum of YT‐iKO mice, we performed image analysis of immunostained brain sections to count proliferating Iba1^+^ (Ki67^+^, Iba1^+^) cells after stroke (Figure 6B and 6C). There was a dramatic increase in proliferating Iba1^+^ cells in CTRL mice, which was statistically significantly attenuated in YT‐iKO mice 3 days after MCAo/reperfusion (Figure 6C). The absolute numbers of Iba1^+^ cells were counted at day 28 after stroke (Figure 6D and 6E). Iba1^+^ cells were significantly increased after MCAo/reperfusion but there was no difference between CTRL and YT‐iKO mice (Figure 6E).

## DISCUSSION

Here, we investigated for the first time the role of endothelial YAP and TAZ in the pathophysiology of ischemic stroke in mice. Our main findings are as follows (1) *Yap*, *Taz* and YAP/TAZ target genes are differentially regulated after stroke. YAP and TAZ protein expression increases in the vasculature during the subacute phase after stroke, with TAZ localized to endothelial cell nuclei, suggesting transient stroke‐induced functional activation of YAP/TAZ in bECs. (2) EC‐specific loss of YAP/TAZ reduces ischemic brain lesion volume without affecting stroke‐induced angiogenesis, suggesting that neuroprotection is mediated by mechanisms other than angiogenesis. (3) YAP/TAZ deficiency induces endothelial transcriptome changes leading to increased myeloid cell recruitment and an anti‐inflammatory perivascular environment in the subacute phase after stroke. (4) We have identified 2 different mechanisms of how YAP/TAZ regulate signaling pathways relevant for bEC function after stroke: cytoplasmic suppression of signal transduction during cGAS‐STING signaling and nuclear transcriptional activation of YAP/TAZ‐TEAD targets from the “alternative Wnt‐YAP/TAZ signaling” pathway. Our findings suggest that YAP and TAZ proteins take part in the inflammatory cascade after stroke. Consequently, modulation of these 2 proteins (or their stroke‐associated signaling pathways) may represent a new treatment strategy toward fostering an anti‐inflammatory and regenerative brain environment after brain ischemia.

### Current Findings on YAP/TAZ Regulation After Ischemic Stroke

To our knowledge, there are only 2 studies that have investigated the regulation of YAP or YAP and TAZ proteins after acute ischemic stroke. Importantly, both studies report contrasting results in the early (24‐hour period) phase after stroke. Gong et al. reported reduced expression of YAP and TAZ in the brain following 90‐minute transient cerebral ischemia in rats whereas the (sub)cellular localization of either protein was not investigated.^50^ In contrast, another study reported increased YAP expression in the nuclei of bECs after 60‐minute MCAo in mice.^51^ The differences in YAP expression between the 2 studies may be due to species differences and the duration of MCA occlusion, affecting lesion maturation as well as cell death dynamics and tissue remodeling.^31^, ^52^, ^53^ Interestingly, antagonizing as well as agonizing pharmaceutical YAP intervention resulted in acute stroke protection.^50^, ^51^ This obvious contradiction highlights the need to better understand cell‐specific YAP/TAZ responses during stroke pathology. By using the 30‐minute MCAo/reperfusion model in mice, we found that cerebral ischemia alters mRNA levels of *Yap*, *Taz*, and YAP/TAZ target genes, with the strongest regulation occurring subacutely. During development, YAP and TAZ interact with TEAD transcription factors, as well as with Notch and BMP (bone morphogenetic protein) signaling in order to regulate EC function.^9^, ^12^, ^13^ Our study confirms that, in the adult mouse brain, the canonical YAP/TAZ–TEAD targets (*Ankrd1*, *Ccn1*, *Ccn2*) are upregulated after MCAo along with *Yap* and *Taz*, whereas the YAP/TAZ‐Notch/BMP target *Hey1* is suppressed. However, in contrast to the previous developmental in vitro data set,^13^ YAP/TAZ‐BMP‐signaling target suppression (ie, suppression of *Id1* and *Smad6*) was not observed after MCAo, indicating the existence of a yet unknown different regulatory mechanism in the ischemic brain. Because gene expression analyses in Figure 1 were performed on bulk ischemic versus sham brain tissue, the spatial localization of YAP/TAZ signaling cannot be attributed to specific brain structures or cell types as *Yap*, *Taz* and their target genes are expressed across multiple cell types in the mouse brain.^54^ Notwithstanding, the differential regulation of YAP/TAZ and their target genes across distinct experimental model systems suggests that the functional roles of YAP/TAZ in the ischemic brain are highly diverse and cell‐type dependent.

### Delayed Response of Endothelial YAP/TAZ After Stroke Mediates Chronic But Not Acute Neuroprotection

To the best of our knowledge, the role of endothelial YAP and TAZ in subacute and chronic ischemic stroke has not been studied before. In order to uncover the spatial distribution of TAZ expression after brain ischemia, we used a *Taz* ^tag^ mouse. Although *Yap*, *Taz*, and YAP/TAZ targets genes are upregulated in ischemic tissue already 24 hours after stroke, we found delayed TAZ protein induction at the subcellular level. Vessel‐associated upregulation and enhanced endothelial nuclear localization of TAZ peaked at 3 days after MCAo/reperfusion, indicating subacute, but not acute, activation of TAZ in bECs after stroke. This delayed TAZ activity was accompanied by distinct YAP expression in the vasculature of ischemic brain tissue 3 days after MCAo and may explain the absence of acute lesion volume differences between YT‐iKO and CTRL animals as measured by MRI. Noninvasive T2w MR imaging is a useful technique for detecting brain injury and is highly sensitive to vasogenic edema.^55^, ^56^ Vasogenic edema develops in the first days after stroke due to progressive endothelial dysfunction, BBB breakdown, and influx of water into tissue.^57^ YAP has been supposed to regulate BBB integrity and cerebral edema via regulation of tight junction protein expression acutely after MCAo.^50^, ^51^ To interfere with YAP activity, the researchers used dexamethasone and verteporfin.^50^, ^51^ It should be noted that neither molecule is EC specific. Considering that YAP/TAZ could also be expressed in other bCs,^54^ the approaches used by the aforementioned studies seem to have some limitations. In our model, we were unable to detect early functional consequences of EC‐specific YAP/TAZ loss that manifest in different MRI lesion volumes. Thus, we conclude that endothelial YAP/TAZ does not contribute to the pathological sequelae leading to endothelial dysfunction and BBB damage in the acute phase of stroke.

### Subacute YAP/TAZ Activity in Endothelial Cells Affects the Inflammatory Brain Environment

Because loss of endothelial YAP/TAZ did not affect EC number, vessel number, and vessel coverage 28 days after 30‐minute MCAo/reperfusion, we believe that neuroprotection in our mouse model is mediated by mechanisms other than angiogenesis. Because EC function in the subacute phase after stroke has been shown to influence BBB characteristics and disease progression in the chronic phase,^58^ we performed a detailed transcriptome analysis in CTRL and YT‐iKO bECs at 3 days after MCAo when YAP/TAZ expression and TAZ activity were highest. The overall transcriptional response of CTRL and YT‐iKO bECs to ischemic stroke was similar to a data set previously published by our group.^38^ However, some important differences between CTRL and YT‐iKO bECs became apparent: we noticed that many of the differentially regulated genes exert their effects extracellularly after stroke and that a large proportion of these genes have receptor regulatory activity and may therefore influence brain inflammatory responses via paracrine mechanisms. This hypothesis is supported by our observation that ischemic YT‐iKO brains display an altered myeloid cell composition relative to CTRL brains, accompanied by a distinct transcriptional inflammatory profile in nonendothelial bCs. The inflammatory profile reflects the heterogeneity of myeloid cells in the ischemic brain, characterized by increased coexpression of infiltrating monocyte markers (*Ccr2*, *Trem1*), anti‐inflammatory markers (*Ccl2*, *Arg1*, *Ym1*), and the BBB–modulating cytokine *IL‐1β*.^42^, ^43^, ^44^, ^45^, ^59^ Concurrently, ischemic YT‐iKO brains exhibit reduced proliferation of Iba1^+^ cells. These findings are consistent with studies supporting the hypothesis that infiltrating monocytes and the suppression of early‐phase microglial proliferation may contribute to neuroprotection.^29^, ^60^, ^61^ We identified YAP/TAZ‐regulated endothelial candidates, including C‐X‐C motif chemokine ligand 10 (*Cxcl10*), bone morphogenetic protein 4 (*Bmp4*), and Gremlin 1 (*Grem1*), all of which are known for their immunomodulatory activity. CXCL10 is a ligand for CXCR3, a G protein‐coupled 7‐transmembrane‐spanning receptor.^62^ This immunoregulatory receptor is expressed on various leukocyte subpopulations in the blood and has been detected in the brain on activated microglia, endothelia, astrocytes, and neurons.^63^, ^64^, ^65^, ^66^ The extent to which EC‐derived CXCL10 contributes to stroke pathology is poorly understood. We and others have demonstrated that brief periods of ischemia (30 minutes) are insufficient to induce *Cxcl10* expression in bECs.^38^, ^67^ Of particular significance is the robust induction of this gene’s expression following YAP/TAZ loss, which correlates with a measurable increase in protein abundance within ischemic brain tissue. Using 1‐hour MCA occlusion periods, Cai et al. showed that EC‐derived CXCL10 limits infarct expansion via increased IL‐10 and ETGF (epidermal growth factor‐like transforming growth factor) expression.^68^ Additionally, CXCL10 can activate microglia and facilitate their CXCR3‐dependent migration.^69^, ^70^, ^71^ In an experimental mouse model of traumatic brain injury, EC‐derived CXCL10 induces a neuroprotective microglia phenotype.^72^ CXCR3^+^/CXCL10 signaling also affects monocyte‐derived and border‐associated macrophages, which play critical roles in blood–brain barrier function and immune cell recruitment after stroke.^29^, ^73^, ^74^, ^75^, ^76^ Of note, in contrast to the upregulation of *Cxcl10*, stroke‐induced expression of *Bmp4* and *Grem1* was abolished in YT‐iKO bECs. A recent study reported that BMP4 polarizes microglia, with BMP4 antagonism exhibiting anti‐inflammatory effects during CNS repair.^77^ Similarly, another study demonstrated that BMP4 induces microglia activation and proliferation.^78^ Therefore, the altered microglia proliferation dynamics and anti‐inflammatory vascular endothelial niche observed in YT‐iKO animals after stroke likely involve targets of the “alternative Wnt–YAP/TAZ signaling” axis. GREM1, a BMP4 antagonist, has been reported to be expressed in endothelial cells subjected to disturbed flow in the mouse aorta and human coronary arteries.^79^, ^80^ Due to its multifunctional roles, GREM1 inhibits monocyte chemotaxis and infiltration by antagonizing macrophage MIF (migration inhibitory factor).^81^, ^82^, ^83^ Therefore, the absence of MCAo‐induced *Grem1* upregulation in YT‐iKO bECs may contribute to the increased monocyte infiltration observed post‐stroke.

### Stroke‐Associated bEC Signaling Cascades Regulated by YAP/TAZ—Nuclear and Cytoplasmic YAP/TAZ Activity Contribute to bEC Function After Stroke

By comparing bECs isolated from CTRL and YT‐iKO mice after MCAo, we found that YAP/TAZ control distinct stroke‐induced pathways and may function not only as regulators of TEAD‐activated gene transcription but also play an important additional cytoplasmic role.

We previously showed that *Bmp4* is upregulated in bECs 3 days after stroke and have reproduced this finding in our current study.^38^ Loss of YAP/TAZ results in failure of the typical MCAo response for *Bmp4* and its transcriptional activator Wnt family member 5B (*Wnt5b*). Both genes are regulated by LATS‐controlled YAP/TAZ activation and nuclear TEAD‐mediated transcription through the alternative Wnt‐YAP/TAZ signaling axis.^84^, ^85^

In addition, our findings uncovered a previously unrecognized regulatory role of YAP/TAZ in modulating cGAS‐STING–mediated *Cxcl10* expression in bECs following stroke. Consistent with previous data sets, we observed increased STING gene and protein levels in MCAo‐bECs and ischemic brain tissue, respectively, serving as indicators of cGAS‐STING pathway activation.^46^, ^67^, ^86^ Activated STING recruits and activates TBK1 and IKK, which subsequently activate IRF3 (interferon regulatory factor 3) and NF‐κB, thereby promoting the transcription of immunoregulatory genes such as *Cxcl10*.^87^ Recent studies have demonstrated that YAP/TAZ directly inhibit cytoplasmic TBK1 kinase activity and are essential for regulating cGAS‐STING signaling in stromal cells.^24^, ^88^ Based on these findings, we propose that YAP/TAZ function as suppressors of cGAS‐STING signaling in the context of stroke. Consequently, loss of YAP/TAZ in bECs relieves this suppression, resulting in increased *Cxcl10* expression. Supporting this hypothesis, we show in vitro that YAP/TAZ knockdown leads to upregulated *Cxcl10* expression.

Our study has some limitations. The extent to which increased EC‐derived CXCL10 or loss of *Wnt5b*, *Bmp4*, and *Grem1* expression in YT‐iKO animals contribute to neuroprotection deserves to be further explored by future studies investigating induced endothelial overexpression or knockout of these proteins in the 30‐minute MCAo model. Although we demonstrated suppressor activity of YAP/TAZ, the specific molecular mechanisms, subcellular localization, and interaction partners remain unclear and require further investigation.

### Conclusions

In summary, we show that YAP/TAZ contributes to bEC function post stroke, which affects long‐term stroke outcome. The subacute transcriptional responses of YAP/TAZ‐deficient bECs create the conditions for a neuroprotective brain environment. Importantly, we newly identified two regulatory pathways associated with YAP/TAZ signaling that affect bEC function and neuroprotective mechanisms after stroke. Our data may support cerebroprotective strategies to treat reperfusion injury or microcirculatory failure even after successful recanalization and provide new evidence for optimal or extended treatment windows after stroke.^89^, ^90^

## Sources of Funding

This work was supported by the Deutsche Forschungsgemeinschaft (Priority Program 2395/GE 2576/6‐1 to Karen Gertz), Germany’s Excellence Strategy (EXC‐2049‐390688087 to Matthias Endres), Clinical Research Group KFO 5023 BeCAUSE‐Y, project 2 EN343/16‐1 (to Matthias Endres), the Bundesministerium für Bildung und Forschung (ERA‐NET Neuron 01EW2304B to Karen Gertz; CSB to Matthias Endres, Karen Gertz, and Golo Kronenberg), the German Center for Neurodegenerative Diseases (DZNE to Matthias Endres), the German Center for Cardiovascular Research (DZHK to Holger Gerhardt, FKZ 81Z0100209 to Matthias Endres and Karen Gertz), the German Center for Mental Health (DZPG to Matthias Endres). Work in the M.P. laboratory was supported by the European Research Council (ERC) Consolidator grant (EMERGE‐773047), the Deutsche Forschungsgemeinschaft (DFG, Project‐ID 456687919—SFB 1531) and the Leducq Foundation.

## Disclosures

None.

## Supporting information

Tables S1 and S2
Figures S1–S6

Unedited Gels
