# Supplementary material for: Loss of Endothelial YAP/TAZ Reduces the Size of Chronic Stroke Lesions and Alters the Endothelial Environment
Source: J Am Heart Assoc. 2025 Nov 26;15(6):e040079. doi: 10.1161/JAHA.124.040079 (PMC13055844; doi:10.1161/JAHA.124.040079)
Supplement: Supplementary file 1 — Tables S1 and S2 Figures S1–S6 [file JAH3-15-e040079-s002.pdf]

# SUPPLEMENTAL MATERIAL

## Table S1. List of primers

### MOUSE

| Gene | for | rev |
|---|---|---|
| *Ankrd1* | CGA GAT ATG CTT GAA TCC ACA GC | CTG TCC TTG GCA TTG AGA TCA G |
| *Arg1* | ATG TGC CCT CTG TCT TTT AGG G | GGT CTC TCA CGT CAT ACT CTG T |
| *Ccl2* | GCT CAG CCA GAT GCA GTT AAC G | GTC AGC ACA GAC CTC TCT CTT G |
| *Ccn1* | CCT TCT CCA CTT GAC CAG ACT G | GTG TTT ACA GTT GGG CTG GAA GC |
| *Ccn2* | CCT CTT CTG CGA TTT CGG CTC C | CTT CCA GTC GGT AGG CAG CTA G |
| *Ccr2* | GAG CCA TAC CTG TAA ATG CCA TG | GCT CAC CAT CAT CGT AGT CAT AC |
| *CD68* | GCA GCA CAG TGG ACA TTC ATG | GAG CAG CCT GTA GCC TTA GAG |
| *Gfap* | GAC AAC TTT GCA CAG GAC CTC G | CCA CTC CTC TGT CTC TTG CAT G |
| *Hey1* | GAG AAG CAG GGA TCT GCT AAG C | GCT CAG ATA ACG GGC AAC TTC G |
| *Id1* | GTC CTG CTC TAC GAC ATG AAC G | GAC TTC AGA CTC CGA GTT CAG C |
| *IL-1b* | CAA CCA ACA AGT GAT ATT CTC CAT G | GAT CCA CAC TCT CCA GCT GCA |
| *IL-6* | GAG GAT ACC ACT CCC AAC AGA CC | AAG TGC ATC ATC GTT GTT CAT ACA |
| *Mrc1* | GTC AGA ACA GAC TGC GTG GA | AGG GAT CGC CTG TTT TCC AG |
| *Smad6* | CAG CAC GCA GTG GAG CTG AAA C | GAC AGA TCC AGT GGC TTG TAC TG |
| *Taz* | TGC TAC AGT GTC CCC ACA AC | TGA CCG GAA TTT TCA CCT GT |
| *Tgm1* | GTG CCC AGA GGA CAT AGT GTA TG | GAC CAT AGT TCC AGG TTC GTT CG |
| *Tnfa* | CCA CCA CGC TCT TCT GTC TA | AGG GTC TGG GCC ATA GAA CT |
| *Tpp2* | CTT CTA TCC AAA GGC TCT CAA GG | CTC TCC AGG TCT CAC CAT CAT G |
| *Trem1* | CTC CTG GTC TTG GAG TCA CTA TC | CAT GTC ATA GCT CCT CCT CAG C |
| *Trem2* | CGT CAC CAT CAC TCT GAA GAA C | GAT CTC CAG CAT CTT GGT CAT C |
| *Yap* | GTC CTC CTT TGA GAT CCC TGA | TGT TGT TGT CTG ATC GTT GTG AT |
| *Ym1* | CTC TAC TCC TCA GAA CCG TCA G | GCA GCC TTG GAA TGT CTT TCT CC |

### HUMAN

| Gene | for | rev |
|---|---|---|
| *CXCL10* | GCA TTC AAG GAG TAC CTC TCT C | GAT GGC CTT CGA TTC TGG ATT C |
| *REEP5* | GAA CTG CAT GAC TGA CCT TCT G | CAG CAA TGC TGA ACA CAC CAT AC |
| *TAZ* | GCA GTA TCC CAG CCA AAT CTC G | CAG CGC ATT GGG CAT ACT CAT G |
| *YAP* | CAG CTC AGC ATC TTC GAC AGT C | CTG TGA CGT TCA TCT GGG ACA G |

## Table S2. List of differentially expressed genes in brain endothelial cells across different contrasts

### Contrast: Sham_YT-iKO_vs_Sham_CTRL_65

| PrimaryID | log2FoldChange | padj | symbol |
|---|---|---|---|
| ENSMUSG00000026579 | -2.19 | 3.12E-25 | F5 |
| ENSMUSG00000032531 | -2.02 | 9.17E-16 | Amotl2 |
| ENSMUSG00000028475 | -1.65 | 1.52E-13 | Spaar |
| ENSMUSG00000012017 | -1.80 | 2.80E-12 | Scarf2 |
| ENSMUSG00000022548 | -2.06 | 1.57E-11 | Apod |
| ENSMUSG00000058966 | -1.96 | 3.39E-10 | Tlcd3b |
| ENSMUSG00000027803 | -1.87 | 1.13E-09 | Wwtr1 |
| ENSMUSG00000084349 | 2.08 | 1.28E-08 | Rpl3-ps1 |
| ENSMUSG00000003617 | -1.84 | 6.43E-08 | Cp |
| ENSMUSG00000021876 | -1.54 | 8.66E-08 | Rnase4 |
| ENSMUSG00000027524 | -1.45 | 1.99E-07 | Edn3 |
| ENSMUSG00000071001 | -1.87 | 1.22E-06 | Hrct1 |
| ENSMUSG00000021256 | -1.69 | 5.03E-06 | Vash1 |
| ENSMUSG00000035407 | -1.24 | 6.61E-06 | Kank4 |
| ENSMUSG00000031465 | -1.38 | 1.01E-05 | Angpt2 |
| ENSMUSG00000049001 | -1.44 | 1.07E-05 | Ndnf |
| ENSMUSG00000020846 | -1.56 | 2.14E-05 | Rflnb |
| ENSMUSG00000053110 | -1.18 | 2.14E-05 | Yap1 |
| ENSMUSG00000047344 | -1.39 | 2.21E-05 | Lancl3 |
| ENSMUSG00000027896 | -1.31 | 3.20E-05 | Slc16a4 |
| ENSMUSG00000021367 | -1.72 | 4.35E-05 | Edn1 |
| ENSMUSG00000032902 | -1.26 | 5.56E-05 | Slc16a1 |
| ENSMUSG00000028194 | -1.16 | 6.46E-05 | Ddah1 |
| ENSMUSG00000031170 | -1.60 | 9.27E-05 | Slc38a5 |
| ENSMUSG00000085071 | -1.39 | 9.88E-05 | |
| ENSMUSG00000072115 | -1.56 | 1.54E-04 | Ang |
| ENSMUSG00000042842 | -1.25 | 1.69E-04 | Serpinb6b |
| ENSMUSG00000053411 | 1.09 | 4.03E-04 | Cbx7 |
| ENSMUSG00000072437 | -1.48 | 4.65E-04 | Nanos1 |
| ENSMUSG00000025479 | -1.63 | 5.63E-04 | Cyp2e1 |
| ENSMUSG00000002228 | 1.27 | 1.19E-03 | Ppm1j |
| ENSMUSG00000038623 | -1.13 | 1.95E-03 | Tm6sf1 |
| ENSMUSG00000074766 | -1.30 | 2.14E-03 | Ism1 |
| ENSMUSG00000024883 | -1.21 | 2.20E-03 | Rin1 |
| ENSMUSG00000110647 | -1.34 | 4.68E-03 | |
| ENSMUSG00000028476 | -0.90 | 6.77E-03 | Reck |
| ENSMUSG00000090863 | -1.12 | 8.21E-03 | |
| ENSMUSG00000029086 | -0.95 | 9.21E-03 | Prom1 |
| ENSMUSG00000047496 | -1.05 | 1.26E-02 | Rnf152 |
| ENSMUSG00000029765 | -0.93 | 1.26E-02 | Plxna4 |
| ENSMUSG00000073802 | -0.86 | 1.26E-02 | Cdkn2b |
| ENSMUSG00000025780 | -0.85 | 1.47E-02 | Itih5 |
| ENSMUSG00000064080 | -0.72 | 1.71E-02 | Fbln2 |
| ENSMUSG00000111531 | -1.18 | 1.72E-02 | Gm32688 |
| ENSMUSG00000022178 | -1.16 | 3.08E-02 | Ajuba |
| ENSMUSG00000036036 | -1.01 | 3.43E-02 | Zfp57 |
| ENSMUSG00000020151 | -0.93 | 4.31E-02 | Ptprr |

| | | | |
|---|---|---|---|
| ENSMUSG00000075010 | -0.87 | 4.55E-02 | AW112010 |
| ENSMUSG00000109243 | -1.12 | 4.62E-02 | |
| ENSMUSG00000041845 | -1.14 | 5.47E-02 | Rhod |
| ENSMUSG00000032359 | -0.81 | 5.63E-02 | Ctsh |
| ENSMUSG00000030170 | -1.09 | 6.52E-02 | Wnt5b |
| ENSMUSG00000041313 | -0.98 | 6.52E-02 | Slc7a1 |
| ENSMUSG00000030770 | -0.92 | 7.28E-02 | Parva |
| ENSMUSG00000048965 | -0.90 | 7.28E-02 | Mrgpre |
| ENSMUSG00000033060 | -0.85 | 7.28E-02 | Lmo7 |
| ENSMUSG00000023348 | -0.79 | 7.28E-02 | Trip6 |
| ENSMUSG00000045954 | -0.76 | 7.28E-02 | Cavin2 |
| ENSMUSG00000032125 | -0.85 | 7.59E-02 | Robo4 |
| ENSMUSG00000037852 | -1.15 | 7.66E-02 | Cpe |
| ENSMUSG00000040562 | -0.97 | 7.70E-02 | Gstm2 |
| ENSMUSG00000041189 | -0.84 | 8.30E-02 | Chrnb1 |
| ENSMUSG00000025887 | -0.87 | 8.33E-02 | Casp12 |
| ENSMUSG00000023903 | -0.90 | 8.65E-02 | Mmp25 |
| ENSMUSG00000051669 | -0.82 | 9.64E-02 | AU021092 |

## Contrast: MCAo_YT-iKO_vs_MCAo_CTRL_78

| PrimaryID | log2FoldChange | padj | symbol |
|---|---|---|---|
| ENSMUSG00000058966 | -2.24 | 7.72E-14 | Tlcd3b |
| ENSMUSG00000020846 | -1.98 | 3.79E-09 | Rflnb |
| ENSMUSG00000032531 | -1.98 | 1.39E-14 | Amotl2 |
| ENSMUSG00000111531 | -1.92 | 1.04E-07 | Gm32688 |
| ENSMUSG00000027803 | -1.92 | 4.41E-10 | Wwtr1 |
| ENSMUSG00000022548 | -1.86 | 1.62E-09 | Apod |
| ENSMUSG00000045573 | -1.84 | 4.10E-03 | Penk |
| ENSMUSG00000053199 | -1.72 | 1.50E-10 | Arhgap20 |
| ENSMUSG00000032902 | -1.64 | 5.71E-09 | Slc16a1 |
| ENSMUSG00000028362 | -1.62 | 2.83E-03 | Tnfsf8 |
| ENSMUSG00000074934 | -1.61 | 4.40E-02 | Grem1 |
| ENSMUSG00000012017 | -1.61 | 1.62E-09 | Scarf2 |
| ENSMUSG00000107479 | -1.60 | 4.10E-03 | 2610300M13Rik |
| ENSMUSG00000026579 | -1.55 | 1.14E-11 | F5 |
| ENSMUSG00000021835 | -1.49 | 2.69E-03 | Bmp4 |
| ENSMUSG00000028600 | -1.48 | 9.56E-03 | Podn |
| ENSMUSG00000071001 | -1.48 | 6.19E-04 | Hrct1 |
| ENSMUSG00000072115 | -1.47 | 7.95E-04 | Ang |
| ENSMUSG00000049001 | -1.41 | 2.39E-05 | Ndnf |
| ENSMUSG00000027896 | -1.37 | 1.21E-05 | Slc16a4 |
| ENSMUSG00000021876 | -1.35 | 9.37E-06 | Rnase4 |
| ENSMUSG00000030170 | -1.34 | 1.70E-03 | Wnt5b |
| ENSMUSG00000028475 | -1.31 | 4.70E-08 | Spaar |
| ENSMUSG00000049241 | -1.30 | 8.44E-02 | Hcar1 |
| ENSMUSG00000042842 | -1.29 | 9.48E-05 | Serpinb6b |
| ENSMUSG00000021256 | -1.28 | 3.92E-03 | Vash1 |
| ENSMUSG00000039004 | -1.27 | 4.20E-03 | Bmp6 |
| ENSMUSG00000028194 | -1.25 | 1.21E-05 | Ddah1 |
| ENSMUSG00000027524 | -1.23 | 6.54E-05 | Edn3 |
| ENSMUSG00000003617 | -1.22 | 5.22E-03 | Cp |
| ENSMUSG00000053110 | -1.21 | 1.26E-05 | Yap1 |

| | | | |
|---|---|---|---|
| ENSMUSG00000110647 | -1.20 | 3.39E-02 | |
| ENSMUSG00000025479 | -1.20 | 9.33E-02 | Cyp2e1 |
| ENSMUSG00000031170 | -1.19 | 2.86E-02 | Slc38a5 |
| ENSMUSG00000045930 | -1.17 | 1.64E-02 | Clec14a |
| ENSMUSG00000028480 | -1.16 | 4.71E-02 | Glipr2 |
| ENSMUSG00000024883 | -1.16 | 5.26E-03 | Rin1 |
| ENSMUSG00000073418 | -1.15 | 2.71E-03 | C4b |
| ENSMUSG00000041313 | -1.13 | 9.10E-03 | Slc7a1 |
| ENSMUSG00000028633 | -1.12 | 4.57E-02 | Ctps |
| ENSMUSG00000021367 | -1.11 | 9.51E-02 | Edn1 |
| ENSMUSG00000035407 | -1.11 | 2.15E-04 | Kank4 |
| ENSMUSG00000020151 | -1.10 | 4.10E-03 | Ptprr |
| ENSMUSG00000073802 | -1.08 | 2.44E-04 | Cdkn2b |
| ENSMUSG00000020660 | -1.08 | 9.56E-03 | Pomc |
| ENSMUSG00000090863 | -1.06 | 2.19E-02 | |
| ENSMUSG00000021892 | -1.05 | 2.37E-02 | Sh3bp5 |
| ENSMUSG00000022797 | -1.05 | 4.57E-02 | Tfrc |
| ENSMUSG00000038623 | -1.01 | 1.59E-02 | Tm6sf1 |
| ENSMUSG00000032359 | -1.00 | 2.71E-03 | Ctsh |
| ENSMUSG00000001493 | -0.99 | 4.57E-02 | Meox1 |
| ENSMUSG00000085071 | -0.99 | 5.53E-02 | |
| ENSMUSG00000047344 | -0.96 | 4.71E-02 | Lancl3 |
| ENSMUSG00000041189 | -0.94 | 2.37E-02 | Chrnb1 |
| ENSMUSG00000047496 | -0.93 | 6.33E-02 | Rnf152 |
| ENSMUSG00000050315 | -0.92 | 3.56E-02 | Synpo2 |
| ENSMUSG00000036611 | -0.91 | 1.33E-02 | Eepd1 |
| ENSMUSG00000030770 | -0.91 | 8.16E-02 | Parva |
| ENSMUSG00000033350 | -0.89 | 8.75E-02 | Chst2 |
| ENSMUSG00000027792 | -0.88 | 4.95E-02 | Bche |
| ENSMUSG00000031209 | -0.86 | 9.60E-02 | Heph |
| ENSMUSG00000024727 | -0.85 | 5.05E-02 | Trpm6 |
| ENSMUSG00000020154 | -0.85 | 2.37E-02 | Ptprb |
| ENSMUSG00000025887 | -0.85 | 9.88E-02 | Casp12 |
| ENSMUSG00000023348 | -0.79 | 6.92E-02 | Trip6 |
| ENSMUSG00000025780 | -0.77 | 5.12E-02 | Itih5 |
| ENSMUSG00000063796 | -0.77 | 8.40E-02 | Slc22a8 |
| ENSMUSG00000023175 | -0.76 | 1.59E-02 | Bsg |
| ENSMUSG00000064080 | -0.75 | 9.56E-03 | Fbln2 |
| ENSMUSG00000029673 | -0.66 | 8.75E-02 | Auts2 |
| ENSMUSG00000026344 | 0.80 | 5.34E-02 | Lypd1 |
| ENSMUSG00000045777 | 0.99 | 9.60E-02 | Ifitm10 |
| ENSMUSG00000053411 | 1.09 | 5.86E-04 | Cbx7 |
| ENSMUSG00000052769 | 1.09 | 8.02E-02 | |
| ENSMUSG00000015568 | 1.64 | 1.33E-02 | Lpl |
| ENSMUSG00000034855 | 1.65 | 2.33E-02 | Cxcl10 |
| ENSMUSG00000084349 | 1.83 | 7.16E-07 | Rpl3-ps1 |
| ENSMUSG00000021091 | 2.73 | 8.75E-02 | Serpina3n |

## Contrast: CTRL_MCAo_vs_CTRL_Sham_203

| PrimaryID | log2FoldChange | padj | symbol |
|---|---|---|---|
| ENSMUSG00000044265 | -1.94 | 6.03E-03 | Olfm5 |
| ENSMUSG00000105909 | -1.74 | 7.09E-04 | |

| | | | |
|---|---|---|---|
| ENSMUSG00000054488 | -1.55 | 6.98E-06 | Gm9946 |
| ENSMUSG00000037362 | -1.36 | 4.54E-02 | Ccn3 |
| ENSMUSG00000030889 | -1.32 | 1.05E-04 | Vwa3a |
| ENSMUSG00000078773 | -1.22 | 1.22E-03 | Rad54b |
| ENSMUSG00000031790 | -1.20 | 2.38E-03 | Mmp15 |
| ENSMUSG00000040998 | -1.11 | 3.23E-02 | Npnt |
| ENSMUSG00000028766 | -1.11 | 5.30E-04 | Alpl |
| ENSMUSG00000040852 | -1.09 | 7.28E-04 | Plekhh2 |
| ENSMUSG00000039405 | -1.08 | 8.47E-03 | Prss23 |
| ENSMUSG00000022371 | -1.08 | 8.65E-04 | Col14a1 |
| ENSMUSG00000006731 | -1.08 | 1.03E-02 | B4galnt1 |
| ENSMUSG00000105707 | -0.99 | 5.01E-02 | |
| ENSMUSG00000085125 | -0.99 | 4.36E-03 | Gm16070 |
| ENSMUSG00000040441 | -0.98 | 6.55E-03 | Slc26a10 |
| ENSMUSG00000004891 | -0.90 | 4.17E-02 | Nes |
| ENSMUSG00000073478 | -0.90 | 1.55E-02 | D730003I15Rik |
| ENSMUSG00000027792 | -0.89 | 1.83E-02 | Bche |
| ENSMUSG00000040488 | -0.88 | 2.59E-03 | Ltbp4 |
| ENSMUSG00000020656 | -0.87 | 1.68E-02 | Grhl1 |
| ENSMUSG00000027524 | -0.87 | 1.82E-02 | Edn3 |
| ENSMUSG00000049690 | -0.81 | 4.50E-02 | Nckap5 |
| ENSMUSG00000041658 | -0.81 | 6.86E-02 | Rragb |
| ENSMUSG00000033715 | -0.81 | 6.99E-02 | Akr1c14 |
| ENSMUSG00000061353 | -0.81 | 3.90E-02 | Cxcl12 |
| ENSMUSG00000110279 | -0.80 | 2.74E-02 | |
| ENSMUSG00000027500 | -0.79 | 9.56E-02 | Stmn2 |
| ENSMUSG00000030621 | -0.78 | 5.55E-02 | Me3 |
| ENSMUSG00000046338 | -0.78 | 7.40E-02 | Gpat2 |
| ENSMUSG00000020019 | -0.78 | 2.34E-02 | Ntn4 |
| ENSMUSG00000021948 | -0.76 | 8.17E-02 | Prkcd |
| ENSMUSG00000022270 | -0.74 | 7.95E-02 | Retreg1 |
| ENSMUSG00000020658 | -0.73 | 4.66E-02 | Efr3b |
| ENSMUSG00000035863 | -0.72 | 5.62E-02 | Palm |
| ENSMUSG00000035407 | -0.71 | 8.17E-02 | Kank4 |
| ENSMUSG00000041362 | -0.71 | 3.97E-02 | Shtn1 |
| ENSMUSG00000029838 | -0.62 | 2.78E-02 | Ptn |
| ENSMUSG00000041836 | 0.75 | 1.93E-02 | Ptpre |
| ENSMUSG00000009621 | 0.75 | 8.17E-02 | Vav2 |
| ENSMUSG00000052861 | 0.79 | 3.41E-03 | Dnah6 |
| ENSMUSG00000026875 | 0.84 | 9.30E-02 | Traf1 |
| ENSMUSG00000005465 | 0.84 | 8.31E-02 | Il27ra |
| ENSMUSG00000025161 | 0.86 | 7.14E-02 | Slc16a3 |
| ENSMUSG00000026193 | 0.86 | 7.14E-02 | Fn1 |
| ENSMUSG00000025492 | 0.86 | 9.81E-02 | Ifitm3 |
| ENSMUSG00000097617 | 0.87 | 8.97E-02 | |
| ENSMUSG00000040111 | 0.87 | 6.06E-02 | Gramd1b |
| ENSMUSG00000031681 | 0.90 | 6.26E-02 | Smad1 |
| ENSMUSG00000027215 | 0.90 | 6.77E-02 | Cd82 |
| ENSMUSG00000034664 | 0.90 | 9.93E-02 | Itga2b |
| ENSMUSG00000041220 | 0.91 | 9.00E-02 | Elovl6 |
| ENSMUSG00000031995 | 0.92 | 8.29E-02 | St14 |
| ENSMUSG00000053175 | 0.95 | 6.02E-02 | Bcl3 |
| ENSMUSG00000021998 | 0.97 | 1.31E-02 | Lcp1 |

| | | | |
|---|---|---|---|
| ENSMUSG00000039116 | 0.99 | 1.01E-02 | Adgrg6 |
| ENSMUSG00000086841 | 1.01 | 4.16E-02 | 2410006H16Rik |
| ENSMUSG00000031103 | 1.01 | 3.58E-02 | Elf4 |
| ENSMUSG00000039232 | 1.02 | 6.81E-02 | Stx11 |
| ENSMUSG00000030790 | 1.02 | 9.07E-02 | Adm |
| ENSMUSG00000004110 | 1.03 | 6.73E-02 | Cacna1e |
| ENSMUSG00000090958 | 1.03 | 6.60E-02 | Lrrc32 |
| ENSMUSG00000028525 | 1.04 | 6.80E-03 | Pde4b |
| ENSMUSG00000025064 | 1.06 | 1.93E-02 | Col17a1 |
| ENSMUSG00000015243 | 1.06 | 8.38E-02 | Abca1 |
| ENSMUSG00000037239 | 1.08 | 8.68E-02 | Spred3 |
| ENSMUSG00000030341 | 1.09 | 6.36E-02 | Tnfrsf1a |
| ENSMUSG00000054855 | 1.09 | 7.34E-02 | Rnd1 |
| ENSMUSG00000021268 | 1.10 | 5.76E-03 | Meg3 |
| ENSMUSG00000022831 | 1.10 | 2.83E-02 | Hcls1 |
| ENSMUSG00000028633 | 1.12 | 2.17E-02 | Ctps |
| ENSMUSG00000020932 | 1.15 | 7.89E-03 | Gfap |
| ENSMUSG00000034586 | 1.15 | 1.15E-02 | Hid1 |
| ENSMUSG00000035673 | 1.15 | 1.88E-02 | Sbno2 |
| ENSMUSG00000022661 | 1.16 | 4.25E-03 | Cd200 |
| ENSMUSG00000026749 | 1.17 | 4.28E-03 | Nek6 |
| ENSMUSG00000002228 | 1.17 | 4.00E-03 | Ppm1j |
| ENSMUSG00000006219 | 1.17 | 1.67E-02 | Fblim1 |
| ENSMUSG00000042784 | 1.20 | 4.54E-02 | Muc1 |
| ENSMUSG00000074227 | 1.20 | 4.65E-02 | Spint2 |
| ENSMUSG00000021453 | 1.20 | 9.13E-02 | Gadd45g |
| ENSMUSG00000041779 | 1.21 | 4.36E-03 | Tram2 |
| ENSMUSG00000024420 | 1.21 | 2.89E-02 | Zfp521 |
| ENSMUSG00000035547 | 1.21 | 6.94E-03 | Capn5 |
| ENSMUSG00000035104 | 1.21 | 4.02E-02 | Eva1a |
| ENSMUSG00000004098 | 1.22 | 4.36E-03 | Col5a3 |
| ENSMUSG00000020484 | 1.23 | 1.91E-02 | Xbp1 |
| ENSMUSG00000034675 | 1.24 | 8.17E-02 | Dbn1 |
| ENSMUSG00000005057 | 1.24 | 3.00E-04 | Sh2b2 |
| ENSMUSG00000003541 | 1.24 | 1.78E-02 | Ier3 |
| ENSMUSG00000050721 | 1.26 | 6.81E-02 | Plekho2 |
| ENSMUSG00000045817 | 1.26 | 1.07E-03 | Zfp36l2 |
| ENSMUSG00000073418 | 1.26 | 2.54E-04 | C4b |
| ENSMUSG00000028970 | 1.26 | 8.65E-04 | Abcb1b |
| ENSMUSG00000055172 | 1.27 | 2.13E-03 | C1ra |
| ENSMUSG00000039005 | 1.27 | 2.55E-04 | Tlr4 |
| ENSMUSG00000033538 | 1.27 | 4.01E-03 | Casp4 |
| ENSMUSG00000036995 | 1.28 | 1.52E-03 | Asap3 |
| ENSMUSG00000030587 | 1.28 | 2.13E-03 | 2200002D01Rik |
| ENSMUSG00000038811 | 1.28 | 1.05E-02 | Gngt2 |
| ENSMUSG00000031375 | 1.29 | 1.03E-02 | Bgn |
| ENSMUSG00000036256 | 1.29 | 1.88E-02 | Igfbp7 |
| ENSMUSG00000035711 | 1.31 | 9.00E-02 | Dok3 |
| ENSMUSG00000026204 | 1.32 | 3.54E-02 | Ptprn |
| ENSMUSG00000023045 | 1.32 | 3.90E-02 | Soat2 |
| ENSMUSG00000030022 | 1.35 | 6.06E-02 | Adamts9 |
| ENSMUSG00000001555 | 1.36 | 6.02E-02 | Fkbp10 |
| ENSMUSG00000027864 | 1.36 | 3.41E-03 | Ptgfrn |

| | | | |
|---|---|---|---|
| ENSMUSG00000030208 | 1.37 | 4.19E-02 | Emp1 |
| ENSMUSG00000028108 | 1.37 | 4.54E-02 | Ecm1 |
| ENSMUSG00000038521 | 1.37 | 7.31E-03 | C1s1 |
| ENSMUSG00000029810 | 1.38 | 3.10E-03 | Tmem176b |
| ENSMUSG00000000253 | 1.39 | 1.10E-04 | Gmpr |
| ENSMUSG00000026223 | 1.39 | 6.84E-03 | Itm2c |
| ENSMUSG00000073599 | 1.39 | 6.55E-03 | Ecscr |
| ENSMUSG00000026890 | 1.40 | 1.91E-02 | Lhx6 |
| ENSMUSG00000031803 | 1.40 | 4.01E-03 | B3gnt3 |
| ENSMUSG00000023991 | 1.40 | 5.51E-03 | Foxp4 |
| ENSMUSG00000045573 | 1.41 | 5.89E-02 | Penk |
| ENSMUSG00000021835 | 1.42 | 3.17E-03 | Bmp4 |
| ENSMUSG00000002897 | 1.42 | 1.01E-03 | Il17ra |
| ENSMUSG00000023224 | 1.43 | 2.13E-03 | Serping1 |
| ENSMUSG00000110618 | 1.43 | 5.48E-02 | Gm39822 |
| ENSMUSG00000026389 | 1.43 | 2.19E-04 | Steap3 |
| ENSMUSG00000049608 | 1.44 | 1.97E-02 | Gpr55 |
| ENSMUSG00000041119 | 1.44 | 1.31E-02 | Pde9a |
| ENSMUSG00000049307 | 1.44 | 3.03E-02 | Fut4 |
| ENSMUSG00000016756 | 1.48 | 1.78E-03 | Cmah |
| ENSMUSG00000056481 | 1.49 | 9.10E-03 | Cd248 |
| ENSMUSG00000029477 | 1.50 | 1.80E-02 | Morn3 |
| ENSMUSG00000097558 | 1.52 | 5.51E-03 | |
| ENSMUSG00000035493 | 1.52 | 7.95E-02 | Tgfbi |
| ENSMUSG00000024661 | 1.53 | 1.39E-03 | Fth1 |
| ENSMUSG00000053199 | 1.56 | 8.47E-09 | Arhgap20 |
| ENSMUSG00000028364 | 1.58 | 3.79E-04 | Tnc |
| ENSMUSG00000032487 | 1.58 | 3.71E-04 | Ptgs2 |
| ENSMUSG00000017446 | 1.60 | 1.49E-02 | C1qtnf1 |
| ENSMUSG00000026822 | 1.60 | 1.97E-02 | Lcn2 |
| ENSMUSG00000052837 | 1.62 | 3.41E-03 | Junb |
| ENSMUSG00000031004 | 1.63 | 4.43E-03 | Mki67 |
| ENSMUSG00000042821 | 1.63 | 5.39E-02 | Snai1 |
| ENSMUSG00000032135 | 1.64 | 4.39E-03 | Mcam |
| ENSMUSG00000002900 | 1.65 | 2.63E-03 | Lamb1 |
| ENSMUSG00000026224 | 1.66 | 1.23E-02 | 4933407L21Rik |
| ENSMUSG00000036606 | 1.67 | 1.69E-04 | Plxnb2 |
| ENSMUSG00000079462 | 1.67 | 7.61E-03 | Gm15737 |
| ENSMUSG00000071714 | 1.67 | 4.25E-03 | Csf2rb2 |
| ENSMUSG00000037852 | 1.68 | 7.08E-05 | Cpe |
| ENSMUSG00000026068 | 1.69 | 1.26E-03 | Il18rap |
| ENSMUSG00000071713 | 1.69 | 2.20E-03 | Csf2rb |
| ENSMUSG00000037418 | 1.70 | 1.26E-05 | Best1 |
| ENSMUSG00000058952 | 1.72 | 3.04E-04 | Cfi |
| ENSMUSG00000044258 | 1.72 | 6.49E-05 | Ctla2a |
| ENSMUSG00000031994 | 1.76 | 7.70E-05 | Adamts8 |
| ENSMUSG00000029082 | 1.76 | 9.68E-05 | Bst1 |
| ENSMUSG00000042485 | 1.77 | 1.28E-02 | Mustn1 |
| ENSMUSG00000027555 | 1.81 | 1.52E-03 | Car13 |
| ENSMUSG00000027368 | 1.82 | 2.50E-04 | Dusp2 |
| ENSMUSG00000030218 | 1.82 | 3.71E-04 | Mgp |
| ENSMUSG00000026475 | 1.85 | 9.37E-08 | Rgs16 |
| ENSMUSG00000048572 | 1.85 | 6.91E-04 | Tmem252 |

| | | | |
|---|---|---|---|
| ENSMUSG00000021190 | 1.90 | 3.04E-04 | Lgmn |
| ENSMUSG00000022346 | 1.91 | 4.36E-03 | Myc |
| ENSMUSG00000005672 | 1.92 | 2.51E-03 | Kit |
| ENSMUSG00000040035 | 1.92 | 3.33E-05 | Disp2 |
| ENSMUSG00000020407 | 1.94 | 1.20E-04 | Upp1 |
| ENSMUSG00000027004 | 1.95 | 9.78E-03 | Frzb |
| ENSMUSG00000091971 | 1.97 | 6.06E-02 | Hspa1a |
| ENSMUSG00000001930 | 1.99 | 3.98E-07 | Vwf |
| ENSMUSG00000027412 | 1.99 | 1.53E-04 | Lpin3 |
| ENSMUSG00000022548 | 2.04 | 1.84E-11 | Apod |
| ENSMUSG00000074934 | 2.05 | 8.65E-04 | Grem1 |
| ENSMUSG00000028600 | 2.06 | 7.19E-06 | Podn |
| ENSMUSG00000017493 | 2.06 | 6.60E-02 | Igfbp4 |
| ENSMUSG00000015745 | 2.09 | 5.02E-05 | Plekho1 |
| ENSMUSG00000023367 | 2.11 | 3.97E-06 | Tmem176a |
| ENSMUSG00000028965 | 2.13 | 3.41E-03 | Tnfrsf9 |
| ENSMUSG00000092557 | 2.16 | 7.23E-02 | |
| ENSMUSG00000005338 | 2.16 | 1.22E-09 | Cadm3 |
| ENSMUSG00000022500 | 2.18 | 3.52E-07 | Litaf |
| ENSMUSG00000019929 | 2.18 | 4.36E-03 | Dcn |
| ENSMUSG00000001131 | 2.19 | 3.00E-02 | Timp1 |
| ENSMUSG00000024349 | 2.37 | 1.24E-09 | Sting1 |
| ENSMUSG00000027221 | 2.41 | 4.94E-07 | Chst1 |
| ENSMUSG00000053113 | 2.42 | 5.55E-08 | Socs3 |
| ENSMUSG00000021922 | 2.43 | 2.92E-02 | Itih4 |
| ENSMUSG00000020681 | 2.59 | 7.19E-08 | Ace |
| ENSMUSG00000021091 | 2.61 | 8.77E-02 | Serpina3n |
| ENSMUSG00000055044 | 2.71 | 1.64E-06 | Pdlim1 |
| ENSMUSG00000026582 | 2.76 | 2.78E-08 | Sele |
| ENSMUSG00000062480 | 2.76 | 1.33E-04 | Acat3 |
| ENSMUSG00000043079 | 2.83 | 2.46E-11 | Synpo |
| ENSMUSG00000025491 | 3.09 | 8.51E-11 | Ifitm1 |
| ENSMUSG00000006403 | 3.09 | 5.65E-05 | Adamts4 |
| ENSMUSG00000028362 | 3.17 | 4.54E-13 | Tnfsf8 |
| ENSMUSG00000051439 | 3.50 | 1.98E-10 | Cd14 |
| ENSMUSG00000050370 | 3.66 | 1.91E-06 | Ch25h |
| ENSMUSG00000037872 | 3.79 | 1.52E-07 | Ackr1 |
| ENSMUSG00000039529 | 3.83 | 5.10E-23 | Atp8b1 |
| ENSMUSG00000064057 | 3.95 | 8.99E-25 | Scgb3a1 |
| ENSMUSG00000026580 | 4.07 | 1.30E-06 | Selp |
| ENSMUSG00000037411 | 4.23 | 7.51E-09 | Serpine1 |
| ENSMUSG00000037095 | 4.59 | 1.98E-10 | Lrg1 |

## Contrast: YT-iKO_MCAo_vs_YT-iKO_Sham_239

| PrimaryID | log2FoldChange | padj | symbol |
|---|---|---|---|
| ENSMUSG00000037362 | -1.96 | 4.90E-05 | Ccn3 |
| ENSMUSG00000054488 | -1.77 | 7.85E-08 | Gm9946 |
| ENSMUSG00000044265 | -1.64 | 6.00E-02 | Olfm5 |
| ENSMUSG00000064833 | -1.55 | 1.59E-02 | Gm25926 |
| ENSMUSG00000086103 | -1.50 | 3.47E-03 | Gm11832 |
| ENSMUSG00000030889 | -1.47 | 6.32E-06 | Vwa3a |
| ENSMUSG00000031790 | -1.44 | 4.75E-05 | Mmp15 |

| | | | |
|---|---|---|---|
| ENSMUSG00000033730 | -1.39 | 2.28E-02 | Egr3 |
| ENSMUSG00000040998 | -1.28 | 4.13E-03 | Npnt |
| ENSMUSG00000078773 | -1.27 | 4.81E-04 | Rad54b |
| ENSMUSG00000107933 | -1.24 | 5.95E-02 | Gm30498 |
| ENSMUSG00000105909 | -1.23 | 5.76E-02 | |
| ENSMUSG00000040852 | -1.16 | 1.55E-04 | Plekhh2 |
| ENSMUSG00000043903 | -1.13 | 6.90E-02 | Zfp469 |
| ENSMUSG00000027875 | -1.09 | 9.25E-02 | Hmgcs2 |
| ENSMUSG00000055116 | -1.06 | 5.14E-02 | Arntl |
| ENSMUSG00000084822 | -1.04 | 5.29E-02 | |
| ENSMUSG00000033715 | -1.03 | 3.41E-03 | Akr1c14 |
| ENSMUSG00000044339 | -1.02 | 6.71E-02 | Alkbh2 |
| ENSMUSG00000027792 | -1.01 | 3.25E-03 | Bche |
| ENSMUSG00000054966 | -0.95 | 5.39E-02 | Lmntd1 |
| ENSMUSG00000049690 | -0.94 | 6.10E-03 | Nckap5 |
| ENSMUSG00000063796 | -0.92 | 3.43E-03 | Slc22a8 |
| ENSMUSG00000041362 | -0.91 | 1.09E-03 | Shtn1 |
| ENSMUSG00000100190 | -0.89 | 6.90E-02 | |
| ENSMUSG00000035000 | -0.89 | 1.44E-03 | Dpp4 |
| ENSMUSG00000015396 | -0.89 | 6.76E-02 | Cd83 |
| ENSMUSG00000040441 | -0.89 | 2.39E-02 | Slc26a10 |
| ENSMUSG00000000805 | -0.88 | 3.24E-02 | Car4 |
| ENSMUSG00000039239 | -0.88 | 7.79E-02 | Tgfb2 |
| ENSMUSG00000037664 | -0.87 | 1.66E-02 | Cdkn1c |
| ENSMUSG00000028766 | -0.86 | 2.41E-02 | Alpl |
| ENSMUSG00000004891 | -0.86 | 6.10E-02 | Nes |
| ENSMUSG00000030589 | -0.83 | 7.64E-02 | Rasgrp4 |
| ENSMUSG00000031147 | -0.81 | 8.05E-02 | Magix |
| ENSMUSG00000022371 | -0.81 | 4.88E-02 | Col14a1 |
| ENSMUSG00000040488 | -0.80 | 9.67E-03 | Ltbp4 |
| ENSMUSG00000050822 | -0.80 | 7.31E-02 | Slc29a4 |
| ENSMUSG00000029838 | -0.78 | 7.97E-04 | Ptn |
| ENSMUSG00000085125 | -0.76 | 7.36E-02 | Gm16070 |
| ENSMUSG00000066842 | -0.75 | 8.30E-02 | Hmcn1 |
| ENSMUSG00000110279 | -0.75 | 5.15E-02 | |
| ENSMUSG00000020658 | -0.74 | 3.98E-02 | Efr3b |
| ENSMUSG00000023175 | -0.73 | 1.29E-02 | Bsg |
| ENSMUSG00000038496 | -0.72 | 5.39E-02 | Slc19a3 |
| ENSMUSG00000024897 | -0.71 | 5.47E-02 | Apba1 |
| ENSMUSG00000020941 | 0.77 | 5.39E-02 | Map3k14 |
| ENSMUSG00000062210 | 0.77 | 9.22E-02 | Tnfaip8 |
| ENSMUSG00000040274 | 0.80 | 3.78E-02 | Cdk6 |
| ENSMUSG00000097534 | 0.83 | 5.94E-02 | Gm16675 |
| ENSMUSG00000025511 | 0.88 | 9.71E-02 | Tspan4 |
| ENSMUSG00000053175 | 0.89 | 9.23E-02 | Bcl3 |
| ENSMUSG00000025855 | 0.89 | 6.59E-02 | Prkar1b |
| ENSMUSG00000030729 | 0.89 | 5.90E-02 | Pgm2l1 |
| ENSMUSG00000026875 | 0.89 | 4.46E-02 | Traf1 |
| ENSMUSG00000025351 | 0.92 | 8.70E-02 | Cd63 |
| ENSMUSG00000035891 | 0.92 | 8.59E-02 | Cerk |
| ENSMUSG00000055978 | 0.92 | 5.39E-02 | Fut2 |
| ENSMUSG00000020038 | 0.92 | 3.72E-02 | Cry1 |
| ENSMUSG00000003617 | 0.92 | 7.65E-02 | Cp |

| | | | |
|---|---|---|---|
| ENSMUSG00000059714 | 0.94 | 5.29E-02 | Flot1 |
| ENSMUSG00000051351 | 0.94 | 8.35E-02 | Zfp46 |
| ENSMUSG00000031995 | 0.94 | 5.73E-02 | St14 |
| ENSMUSG00000041220 | 0.94 | 5.95E-02 | Elovl6 |
| ENSMUSG00000054008 | 0.94 | 7.09E-02 | Ndst1 |
| ENSMUSG00000056737 | 0.96 | 5.59E-02 | Capg |
| ENSMUSG00000025064 | 0.96 | 4.57E-02 | Col17a1 |
| ENSMUSG00000035778 | 0.96 | 4.43E-02 | Ggta1 |
| ENSMUSG00000026544 | 0.97 | 9.88E-02 | Dusp23 |
| ENSMUSG00000045934 | 0.97 | 9.27E-02 | Mtmr11 |
| ENSMUSG00000062300 | 0.97 | 4.60E-02 | Nectin2 |
| ENSMUSG00000031616 | 0.98 | 5.39E-02 | Ednra |
| ENSMUSG00000079037 | 1.01 | 7.65E-02 | Prnp |
| ENSMUSG00000026072 | 1.03 | 9.22E-02 | Il1r1 |
| ENSMUSG00000053137 | 1.03 | 7.78E-02 | Mapk11 |
| ENSMUSG00000034586 | 1.04 | 3.78E-02 | Hid1 |
| ENSMUSG00000022508 | 1.04 | 7.65E-03 | Bcl6 |
| ENSMUSG00000020901 | 1.04 | 2.92E-02 | Pik3r5 |
| ENSMUSG00000051341 | 1.05 | 8.19E-02 | Zfp52 |
| ENSMUSG00000042363 | 1.05 | 8.59E-02 | Lgalsl |
| ENSMUSG00000030134 | 1.05 | 7.98E-03 | Rasgef1a |
| ENSMUSG00000039005 | 1.05 | 6.60E-03 | Tlr4 |
| ENSMUSG00000040711 | 1.06 | 5.54E-02 | Sh3pxd2b |
| ENSMUSG00000055172 | 1.06 | 2.02E-02 | C1ra |
| ENSMUSG00000079020 | 1.06 | 9.05E-03 | Slc45a4 |
| ENSMUSG00000005397 | 1.06 | 4.64E-02 | Nid1 |
| ENSMUSG00000020689 | 1.08 | 1.86E-02 | Itgb3 |
| ENSMUSG00000048612 | 1.09 | 3.16E-02 | Myof |
| ENSMUSG00000021939 | 1.09 | 6.16E-02 | Ctsb |
| ENSMUSG00000021903 | 1.10 | 3.65E-02 | Galnt15 |
| ENSMUSG00000048120 | 1.10 | 1.49E-02 | Entpd1 |
| ENSMUSG00000004110 | 1.10 | 3.16E-02 | Cacna1e |
| ENSMUSG00000020262 | 1.11 | 1.94E-02 | Adarb1 |
| ENSMUSG00000032412 | 1.11 | 3.32E-02 | Atp1b3 |
| ENSMUSG00000030790 | 1.12 | 3.67E-02 | Adm |
| ENSMUSG00000026870 | 1.13 | 3.34E-02 | Cutal |
| ENSMUSG00000028364 | 1.13 | 3.78E-02 | Tnc |
| ENSMUSG00000041779 | 1.13 | 1.09E-02 | Tram2 |
| ENSMUSG00000040612 | 1.13 | 3.34E-02 | Ildr2 |
| ENSMUSG00000021367 | 1.14 | 3.78E-02 | Edn1 |
| ENSMUSG00000011305 | 1.15 | 9.71E-02 | Plin5 |
| ENSMUSG00000037071 | 1.15 | 1.41E-02 | Scd1 |
| ENSMUSG00000036995 | 1.15 | 6.60E-03 | Asap3 |
| ENSMUSG00000024754 | 1.16 | 2.11E-02 | Cemip2 |
| ENSMUSG00000054793 | 1.16 | 4.19E-02 | Cadm4 |
| ENSMUSG00000031016 | 1.16 | 5.20E-04 | Wee1 |
| ENSMUSG00000026389 | 1.16 | 7.60E-03 | Steap3 |
| ENSMUSG00000017756 | 1.17 | 9.24E-03 | Slc12a7 |
| ENSMUSG00000022661 | 1.17 | 3.36E-03 | Cd200 |
| ENSMUSG00000026193 | 1.17 | 1.09E-03 | Fn1 |
| ENSMUSG00000034390 | 1.17 | 1.04E-02 | Cmip |
| ENSMUSG00000022816 | 1.18 | 9.20E-02 | Fstl1 |
| ENSMUSG00000097558 | 1.21 | 5.39E-02 | |

| | | | |
|---|---|---|---|
| ENSMUSG00000000957 | 1.21 | 6.67E-02 | Mmp14 |
| ENSMUSG00000023045 | 1.22 | 5.39E-02 | Soat2 |
| ENSMUSG00000026773 | 1.24 | 3.25E-03 | Pfkfb3 |
| ENSMUSG00000024087 | 1.25 | 3.58E-02 | Cyp1b1 |
| ENSMUSG00000028525 | 1.25 | 2.27E-04 | Pde4b |
| ENSMUSG00000037820 | 1.25 | 7.98E-03 | Tgm2 |
| ENSMUSG00000028362 | 1.27 | 6.90E-02 | Tnfsf8 |
| ENSMUSG00000032966 | 1.27 | 6.90E-02 | Fkbp1a |
| ENSMUSG00000028970 | 1.27 | 3.36E-04 | Abcb1b |
| ENSMUSG00000033538 | 1.27 | 3.63E-03 | Casp4 |
| ENSMUSG00000005413 | 1.28 | 4.31E-02 | Hmox1 |
| ENSMUSG00000000555 | 1.28 | 1.38E-02 | Itga5 |
| ENSMUSG00000036256 | 1.32 | 1.36E-02 | Igfbp7 |
| ENSMUSG00000026365 | 1.32 | 1.65E-03 | Cfh |
| ENSMUSG00000073489 | 1.33 | 1.26E-02 | Ifi204 |
| ENSMUSG00000052310 | 1.33 | 6.59E-03 | Slc39a1 |
| ENSMUSG00000031375 | 1.34 | 6.31E-03 | Bgn |
| ENSMUSG00000019590 | 1.34 | 2.08E-03 | Cyb561 |
| ENSMUSG00000026224 | 1.35 | 7.67E-02 | 4933407L21Rik |
| ENSMUSG00000022122 | 1.36 | 5.39E-02 | Ednrb |
| ENSMUSG00000035673 | 1.37 | 1.34E-03 | Sbno2 |
| ENSMUSG00000085427 | 1.37 | 1.76E-02 | 6430710C18Rik |
| ENSMUSG00000034684 | 1.38 | 3.94E-02 | Sema3f |
| ENSMUSG00000034675 | 1.38 | 2.22E-02 | Dbn1 |
| ENSMUSG00000020810 | 1.39 | 6.90E-02 | Cygb |
| ENSMUSG00000054855 | 1.39 | 4.21E-03 | Rnd1 |
| ENSMUSG00000004328 | 1.40 | 2.73E-02 | Hif3a |
| ENSMUSG00000036478 | 1.40 | 2.63E-03 | Btg1 |
| ENSMUSG00000030341 | 1.40 | 2.16E-03 | Tnfrsf1a |
| ENSMUSG00000052837 | 1.40 | 2.22E-02 | Junb |
| ENSMUSG00000027555 | 1.40 | 3.32E-02 | Car13 |
| ENSMUSG00000035104 | 1.41 | 3.17E-03 | Eva1a |
| ENSMUSG00000015243 | 1.41 | 2.26E-03 | Abca1 |
| ENSMUSG00000037239 | 1.42 | 2.93E-03 | Spred3 |
| ENSMUSG00000015568 | 1.43 | 3.21E-02 | Lpl |
| ENSMUSG00000106062 | 1.44 | 6.74E-03 | |
| ENSMUSG00000003541 | 1.44 | 1.76E-03 | Ier3 |
| ENSMUSG00000039911 | 1.45 | 7.98E-03 | Spsb1 |
| ENSMUSG00000044258 | 1.46 | 1.61E-03 | Ctla2a |
| ENSMUSG00000058952 | 1.46 | 3.47E-03 | Cfi |
| ENSMUSG00000001555 | 1.46 | 2.53E-02 | Fkbp10 |
| ENSMUSG00000085148 | 1.46 | 1.49E-02 | Mir22hg |
| ENSMUSG00000027368 | 1.47 | 4.99E-03 | Dusp2 |
| ENSMUSG00000053626 | 1.47 | 1.44E-03 | Tll1 |
| ENSMUSG00000039232 | 1.48 | 2.08E-04 | Stx11 |
| ENSMUSG00000037211 | 1.49 | 1.10E-02 | Spry1 |
| ENSMUSG00000054435 | 1.55 | 2.10E-04 | Gimap4 |
| ENSMUSG00000073599 | 1.55 | 1.09E-03 | Ecscr |
| ENSMUSG00000045817 | 1.56 | 6.32E-06 | Zfp36l2 |
| ENSMUSG00000029084 | 1.56 | 4.96E-04 | Cd38 |
| ENSMUSG00000024661 | 1.56 | 8.56E-04 | Fth1 |
| ENSMUSG00000024420 | 1.56 | 4.96E-04 | Zfp521 |
| ENSMUSG00000023224 | 1.56 | 2.17E-04 | Serping1 |

| | | | |
|---|---|---|---|
| ENSMUSG00000029082 | 1.59 | 1.44E-03 | Bst1 |
| ENSMUSG00000031803 | 1.62 | 2.19E-04 | B3gnt3 |
| ENSMUSG00000055866 | 1.62 | 9.79E-04 | Per2 |
| ENSMUSG00000002897 | 1.63 | 3.42E-05 | Il17ra |
| ENSMUSG00000028108 | 1.63 | 4.19E-03 | Ecm1 |
| ENSMUSG00000027864 | 1.64 | 8.15E-05 | Ptgfrn |
| ENSMUSG00000026223 | 1.65 | 3.14E-04 | Itm2c |
| ENSMUSG00000042485 | 1.66 | 2.41E-02 | Mustn1 |
| ENSMUSG00000005338 | 1.71 | 9.93E-06 | Cadm3 |
| ENSMUSG00000020676 | 1.71 | 1.86E-02 | Ccl11 |
| ENSMUSG00000026475 | 1.72 | 1.56E-06 | Rgs16 |
| ENSMUSG00000068220 | 1.72 | 2.02E-02 | Lgals1 |
| ENSMUSG00000001473 | 1.78 | 3.19E-03 | Tubb6 |
| ENSMUSG00000036606 | 1.79 | 2.59E-05 | Plxnb2 |
| ENSMUSG00000004371 | 1.79 | 2.53E-02 | Il11 |
| ENSMUSG00000032548 | 1.80 | 2.93E-03 | Slco2a1 |
| ENSMUSG00000032487 | 1.81 | 1.28E-05 | Ptgs2 |
| ENSMUSG00000020484 | 1.81 | 1.24E-05 | Xbp1 |
| ENSMUSG00000002900 | 1.83 | 3.25E-04 | Lamb1 |
| ENSMUSG00000029810 | 1.83 | 6.19E-06 | Tmem176b |
| ENSMUSG00000035493 | 1.84 | 7.09E-03 | Tgfbi |
| ENSMUSG00000032135 | 1.90 | 2.71E-04 | Mcam |
| ENSMUSG00000027412 | 1.93 | 2.37E-04 | Lpin3 |
| ENSMUSG00000056481 | 1.94 | 6.91E-05 | Cd248 |
| ENSMUSG00000030022 | 1.95 | 2.33E-04 | Adamts9 |
| ENSMUSG00000030748 | 1.96 | 1.48E-04 | Il4ra |
| ENSMUSG00000029762 | 1.96 | 1.23E-02 | Akr1b8 |
| ENSMUSG00000040035 | 1.96 | 1.24E-05 | Disp2 |
| ENSMUSG00000042821 | 1.98 | 3.72E-03 | Snai1 |
| ENSMUSG00000021190 | 1.98 | 1.13E-04 | Lgmn |
| ENSMUSG00000027221 | 2.00 | 1.13E-04 | Chst1 |
| ENSMUSG00000079462 | 2.05 | 1.55E-04 | Gm15737 |
| ENSMUSG00000087006 | 2.07 | 7.98E-03 | Gm13889 |
| ENSMUSG00000019929 | 2.09 | 7.59E-03 | Dcn |
| ENSMUSG00000030218 | 2.09 | 1.24E-05 | Mgp |
| ENSMUSG00000015745 | 2.10 | 3.03E-05 | Plekho1 |
| ENSMUSG00000055044 | 2.10 | 7.20E-04 | Pdlim1 |
| ENSMUSG00000048572 | 2.12 | 2.58E-05 | Tmem252 |
| ENSMUSG00000024349 | 2.13 | 1.15E-07 | Sting1 |
| ENSMUSG00000022500 | 2.16 | 4.81E-07 | Litaf |
| ENSMUSG00000020407 | 2.22 | 1.40E-06 | Upp1 |
| ENSMUSG00000022548 | 2.23 | 9.80E-14 | Apod |
| ENSMUSG00000048636 | 2.24 | 4.65E-05 | A730049H05Rik |
| ENSMUSG00000037852 | 2.30 | 8.83E-10 | Cpe |
| ENSMUSG00000050234 | 2.30 | 9.93E-06 | Gja4 |
| ENSMUSG00000001930 | 2.32 | 6.34E-10 | Vwf |
| ENSMUSG00000005672 | 2.36 | 2.76E-05 | Kit |
| ENSMUSG00000034855 | 2.38 | 1.13E-05 | Cxcl10 |
| ENSMUSG00000001131 | 2.40 | 4.12E-03 | Timp1 |
| ENSMUSG00000092557 | 2.45 | 1.44E-02 | |
| ENSMUSG00000023367 | 2.53 | 4.10E-09 | Tmem176a |
| ENSMUSG00000025491 | 2.54 | 2.15E-07 | Ifitm1 |
| ENSMUSG00000062480 | 2.55 | 2.52E-04 | Acat3 |

| | | | |
|---|---|---|---|
| ENSMUSG00000029163 | 2.56 | 1.82E-03 | Emilin1 |
| ENSMUSG00000017493 | 2.59 | 3.35E-03 | Igfbp4 |
| ENSMUSG00000021922 | 2.71 | 6.16E-03 | Itih4 |
| ENSMUSG00000043079 | 2.72 | 1.58E-10 | Synpo |
| ENSMUSG00000026822 | 2.77 | 6.30E-08 | Lcn2 |
| ENSMUSG00000029304 | 2.94 | 1.68E-02 | Spp1 |
| ENSMUSG00000006403 | 2.97 | 9.70E-05 | Adamts4 |
| ENSMUSG00000026582 | 3.07 | 4.55E-11 | Sele |
| ENSMUSG00000064057 | 3.16 | 1.90E-15 | Scgb3a1 |
| ENSMUSG00000020681 | 3.16 | 4.66E-12 | Ace |
| ENSMUSG00000051439 | 3.22 | 6.27E-09 | Cd14 |
| ENSMUSG00000024903 | 3.28 | 1.83E-03 | Lao1 |
| ENSMUSG00000039529 | 3.39 | 7.54E-18 | Atp8b1 |
| ENSMUSG00000026580 | 3.67 | 2.42E-05 | Selp |
| ENSMUSG00000053113 | 3.72 | 1.79E-19 | Socs3 |
| ENSMUSG00000037872 | 3.77 | 1.49E-07 | Ackr1 |
| ENSMUSG00000050370 | 3.97 | 1.15E-07 | Ch25h |
| ENSMUSG00000037411 | 4.30 | 2.12E-09 | Serpine1 |
| ENSMUSG00000021091 | 4.38 | 1.03E-05 | Serpina3n |
| ENSMUSG00000037095 | 4.86 | 7.28E-12 | Lrg1 |

**Figure S1. Characterization of tamoxifen treated CTRL and YT-iKO mice 3 days after sham surgery.**

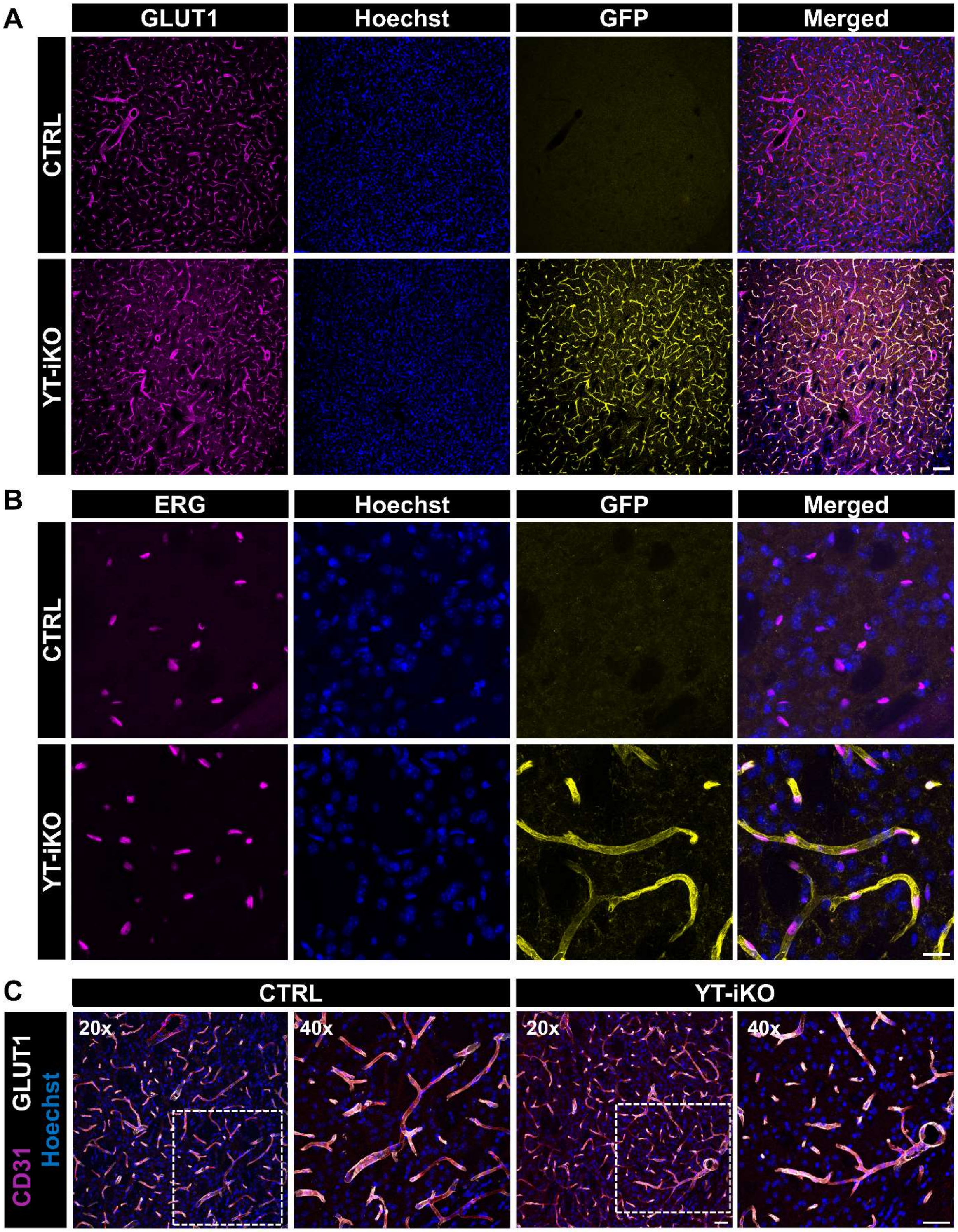

**(A), (B)** Endothelial cell specificity of transgene expression. **(A)** Distinct vascular eGFP reporter signal in the brain of adult YT-iKO (*Pdgfb-iCreER-eGFP*$^{TG/+}$*Yap*$^{fl/fl}$*Taz*$^{fl/fl}$), but not CTRL (*Pdgfb-iCreER-eGFP*$^{+/+}$*Yap*$^{fl/fl}$*Taz*$^{fl/fl}$) mice. The eGFP signal (yellow) co-localizes with the endothelial cell marker GLUT1 (magenta). No eGFP expression was observed outside the vasculature. Scale bar: 100 µm. **(B)** EGFP signal (yellow) co-localizes with the endothelial cell marker ERG (magenta). Scale bar: 20 µm. **(C)** CD31$^{+}$ (magenta) and GLUT1$^{+}$ (grays) vascular structures in the YT-iKO brain show no obvious vascular malformation in comparison to the CTRL brain. Scale bar: 40 µm. Representative images acquired by confocal microscopy **(A-C)** were taken from the lateral striatum as z-stacks and visualized as maximum intensity projections. Hoechst (blue) as nuclear counterstain.

**Figure S2. Characterization of tamoxifen-treated CTRL and YT-iKO mice 3 days after MCAo.**

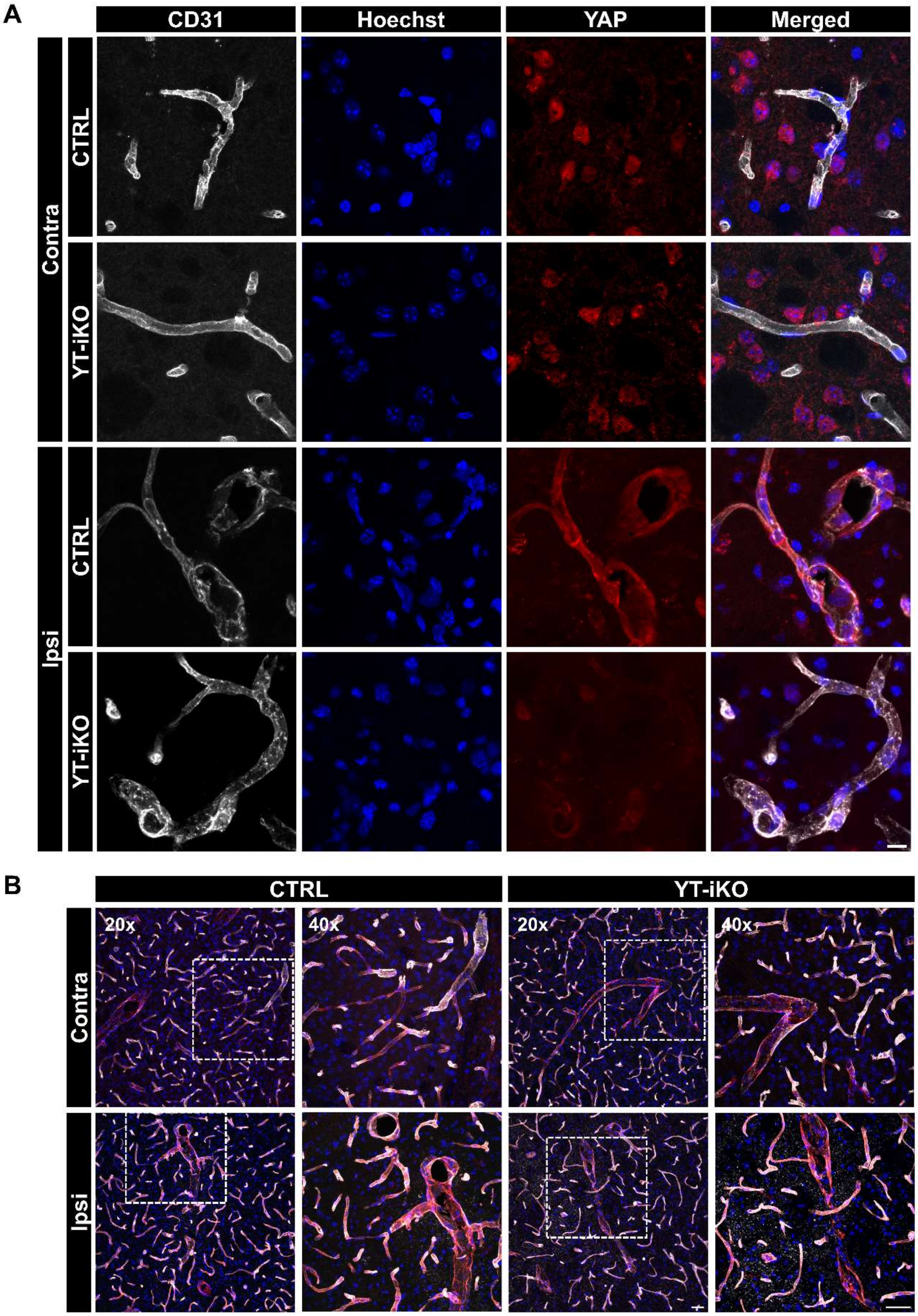

**(A)** Downregulation of YAP expression (red) in $CD31^+$ vessels (grays) in the ipsilateral striatum of a YT-iKO mouse. Please note that YAP expression is upregulated in the vasculature 3 days after MCAo, whereas no $YAP^+$ vessels were detected in the contralateral striatum. Scale bar: 10 µm. **(B)** $CD31^+$ (magenta) and $GLUT1^+$ (grays) vascular structures in the YT-iKO brain show no obvious vascular abnormalities in comparison to the CTRL brain. Scale bar: 40µm. Representative images acquired by confocal microscopy **(A, B)** were taken from the lateral striatum as z-stacks and visualized as maximum intensity projections. Hoechst (blue) as nuclear counterstain. Contra: contralateral striatum, Ipsi: ipsilateral striatum. GLUT1, facilitated glucose transporter member 1; CD31, platelet endothelial cell adhesion molecule.

**Figure S3. Immunohistochemistry of tamoxifen-treated CTRL and YT-iKO mice 28 days after MCAo.**

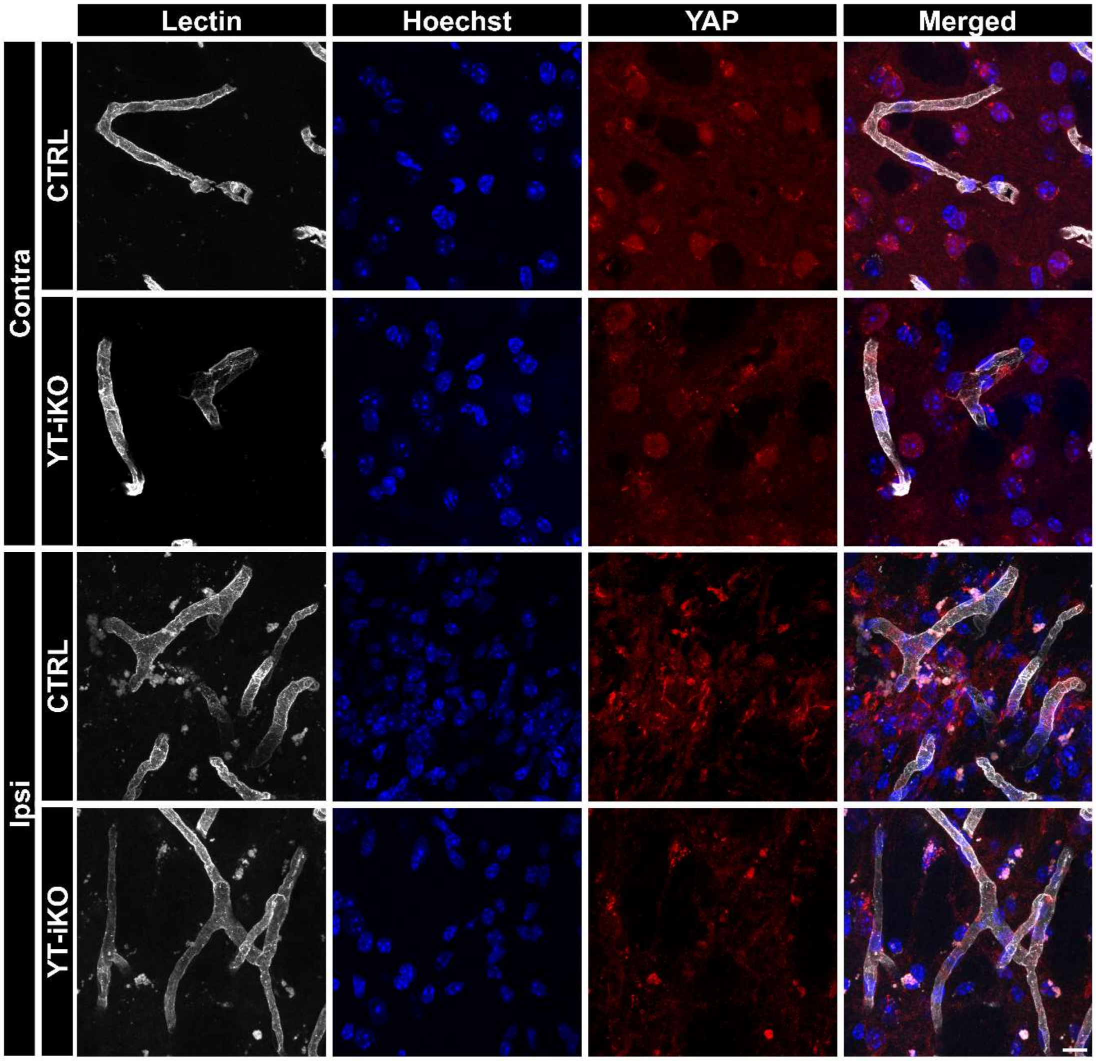


Lectin-perfused vasculature (grays) in the lateral striatum of CTRL and YT-iKO mice. In the ipsilateral striatum, YAP (red) is no longer expressed at detectable levels in the vasculature of CTRL mice (cf. upregulated *Yap* expression at acute to subacute stroke time points in Figure 1 and increased YAP immunostaining in the vasculature in Figure S2). Representative images acquired by confocal microscopy were taken from the lateral striatum as z-stacks and visualized as maximum intensity projections. Hoechst (blue) as nuclear counterstain. Contra: contralateral striatum, Ipsi: ipsilateral striatum. Scale bar: 10 µm.

**Figure S4. RNA analysis of CD31⁺ brain endothelial cells (bECs) and CD31⁻ brain cells (bCs) 3 days after MCAo.**

A

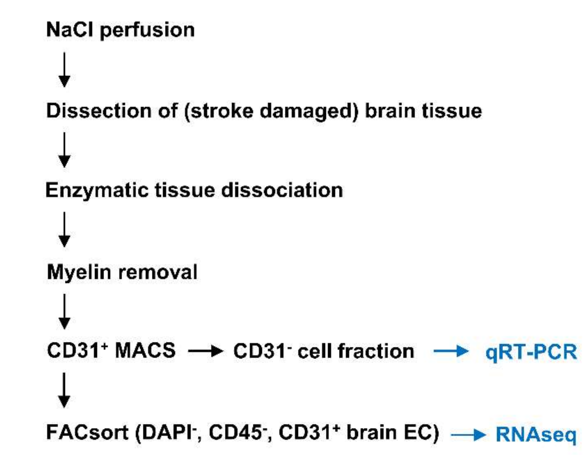


B

| Sample | Genotype | Treatment | Number of mice |
|---|---|---|---|
| 1 | CTRL | MCAo | 3 |
| 2 | CTRL | MCAo | 3 |
| 3 | CTRL | MCAo | 3 |
| 4 | CTRL | MCAo | 2 |
| 1 | CTRL | Sham | 2 |
| 2 | CTRL | Sham | 2 |
| 3 | CTRL | Sham | 2 |
| 4 | CTRL | Sham | 2 |
| 1 | YT-iKO | MCAo | 3 |
| 2 | YT-iKO | MCAo | 3 |
| 3 | YT-iKO | MCAo | 2 |
| 4 | YT-iKO | MCAo | 4 |
| 1 | YT-iKO | Sham | 2 |
| 2 | YT-iKO | Sham | 2 |
| 3 | YT-iKO | Sham | 2 |
| 4 | YT-iKO | Sham | 2 |

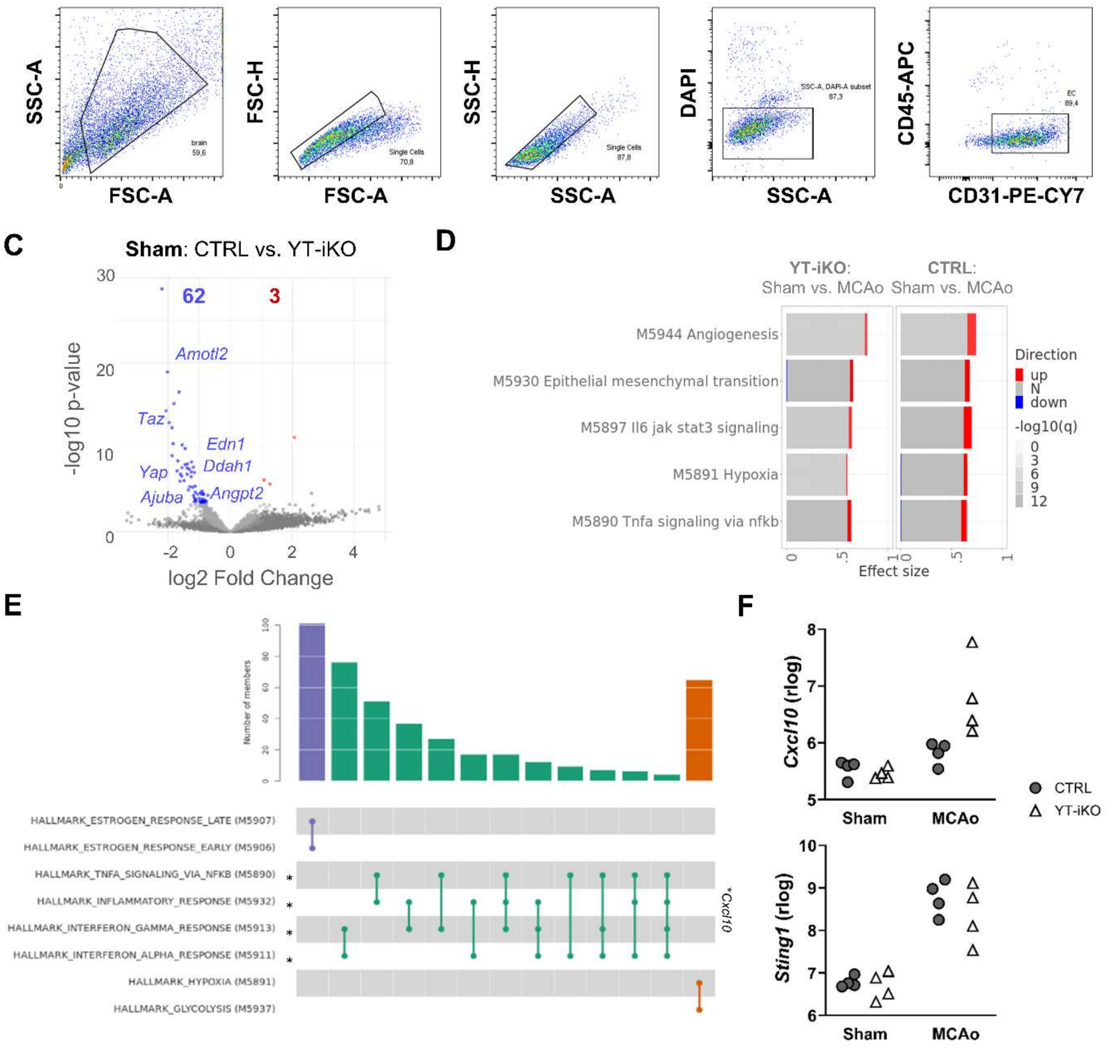

**(A)** Overview and sorting strategy (including FACS gating) for the isolation of brain endothelialcells (bECs) and non-endothelial brain cells (bCs). **(B)** Information on the number of mice used in the RNA analysis experiment. Brain tissue of 2-4 mice was pooled for each replicate. **(C)** Volcano plot depicting differentially expressed genes (DEG) between CTRL bECs and YT-iKO bECs from sham-operated mice. Downregulated expression of *Yap*, *Taz* and YAP/TAZ targets (*Ajuba*, *Amotl2*, *Angpt2*, *Ddah1*, *Edn1*) indicates successful Cre-loxP recombination after tamoxifen injections. **(D)** Panel plot depicts the result of the GSA for CTRL and YT-iKO bECs after MCAo. Row corresponds to the significantly enriched molecular signature database term (hallmark), column corresponds to contrast, bar length indicates effect size (AUC, area under curve), intensity of the color corresponds to the FDR, red and blue fragments indicate fraction of differently expressed genes within the contrast (padj < 0.1). **(E)** Upset plot depicts the gene overlap between significantly enriched terms derived from the GSA for the contrast MCAo: YT-iKO versus CTRL. **(F)** Regularized log transformed expression (rlog) of *Cxcl10* and its upstream activator *Sting1* (stimulator of interferon response cGAMP interactor 1) in bECs. DEG analyses (DEseq2) revealed *Cxcl10* upregulation only in YT-iKO bECs after MCAo (*p*adj = 1.1e-5), while *Sting1* is upregulated after MCAO in both CTRL and YT-iKO bECs (CTRL: *p*adj = 1.2e-9; YT-iKO: *p*adj = 1.1e-7).

**Figure S5. FACS gating strategies for blood and brain cells 3 days after MCAo.**

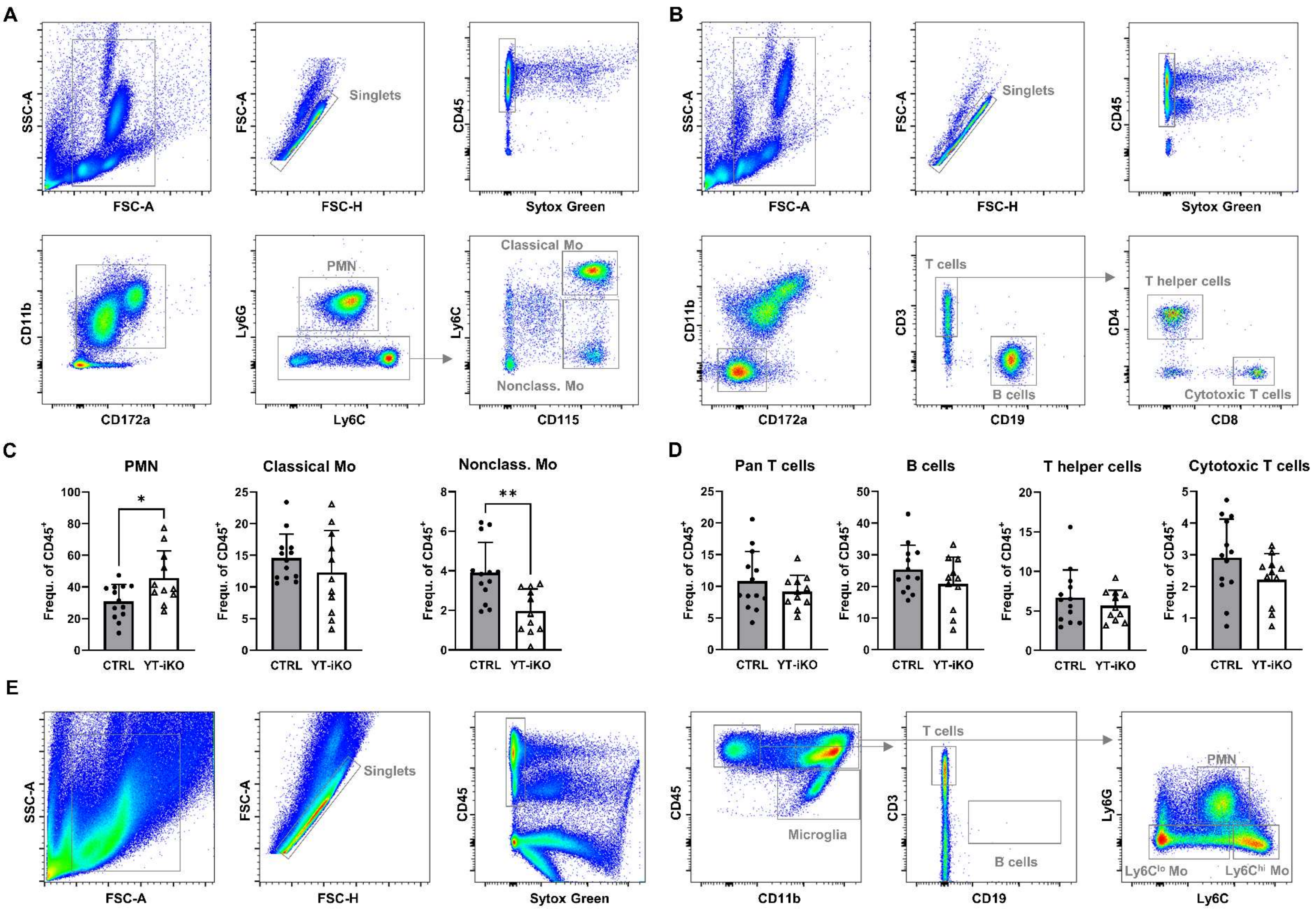

**(A)** Gating strategy of blood myeloid cells. Neutrophils (PMN) were defined as Sytox green$^{-}$, CD45$^{+}$, CD11b$^{+}$, CD172$^{+}$, Ly6G$^{+}$. Ly6C$^{hi}$ classical and Ly6C$^{lo}$ nonclassical monocytes (Mo) were defined as Sytox green$^{-}$, CD45$^{+}$, CD11b$^{+}$, CD172$^{+}$, Ly6G$^{-}$, CD115$^{+}$. **(B)** Gating strategy of blood lymphocytes (Sytox green$^{-}$, CD45$^{+}$, CD11b$^{-}$, CD172$^{-}$). B-cells were identified by CD19 (CD19$^{+}$, CD3$^{-}$). T cells (CD3$^{+}$, CD19$^{-}$) were further seperated into CD4$^{+}$ T helper cells and CD8$^{+}$ cytotoxic T cells. **(C)** Frequency of blood myeloid cell populations (neutrophils, classical and nonclassical monocytes) in CTRL and YT-iKO mice. Mean ± SD, CTRL n = 13, YT-iKO n = 11, unpaired t test, *$p < .05$, **$p < .01$. **(D)** Frequency of blood lymphocyte populations (T cells, B cells, T helper cells, cytotoxic T cells) in CTRL and YT-iKO mice. Mean ± SD, CTRL n = 13, YT-iKO n = 11, unpaired t test. **(E)** Gating strategy for brain immune cell populations. Microglia were defined as Sytox green$^{-}$, CD45$^{lo}$, CD11b$^{+}$. T cells were defined as Sytox green$^{-}$, CD45$^{hi}$, CD11b$^{-}$, CD3$^{+}$, CD19$^{-}$ and B cells as Sytox green$^{-}$, CD45$^{hi}$, CD11b$^{-}$, CD3$^{-}$, CD19$^{+}$. Invading myeloid cells (Sytox green$^{-}$, CD45$^{hi}$, CD11b$^{+}$) were further gated based on Ly6G$^{+}$ (PMN), Ly6C$^{hi}$ (classical monocytes) and Ly6C$^{lo}$ (nonclassical monocytes) marker expression.

**Figure S6. Immunostaining for neutrophils and T cells in the brain of CTRL and YT-iKO mice 3 days after MCAo.**

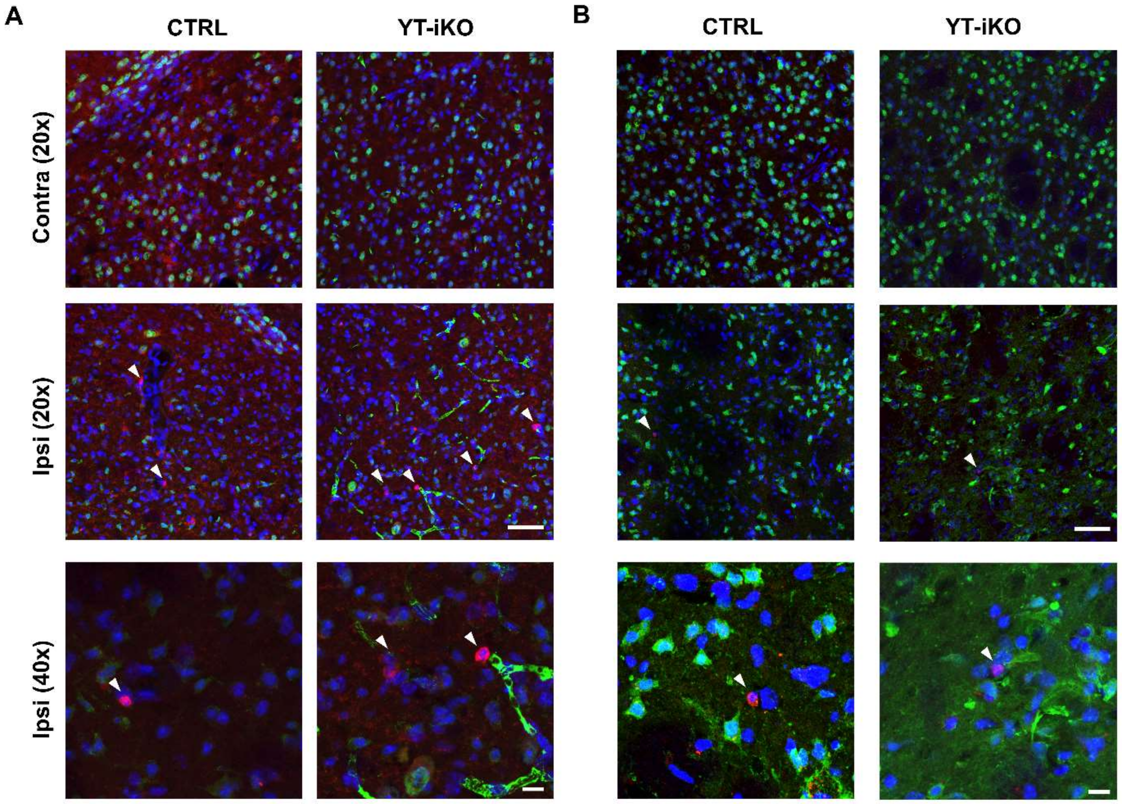


Ly6G⁺ cells (neutrophils, shown in red) were only detected in the ipsilateral hemisphere. **(B)** CD3⁺ cells (T cells, red) were rarely found in the ischemic hemisphere. **(A, B)** Representative images acquired by confocal microscopy were taken from the lateral striatum as z-stacks and visualized as maximum intensity projections. Hoechst (blue) as nuclear counterstain. NeuN (green) as a marker for neuronal loss in the ipsilateral striatum. Additionally, YT-iKO mice show endogenous GFP expression in the vasculature. Contra: contralateral striatum, Ipsi: ipsilateral striatum. Scale bars: 50 µm for images taken with 20x objective, 10 µm for images taken with 40x objective.
