# Supplementary figures and images for: Loss of Endothelial YAP/TAZ Reduces the Size of Chronic Stroke Lesions and Alters the Endothelial Environment
Source: J Am Heart Assoc. 2025 Nov 26;15(6):e040079. doi: 10.1161/JAHA.124.040079 (PMC13055844; doi:10.1161/JAHA.124.040079)

## UNEDITED GELS

Full unedited blots for Figure 5B

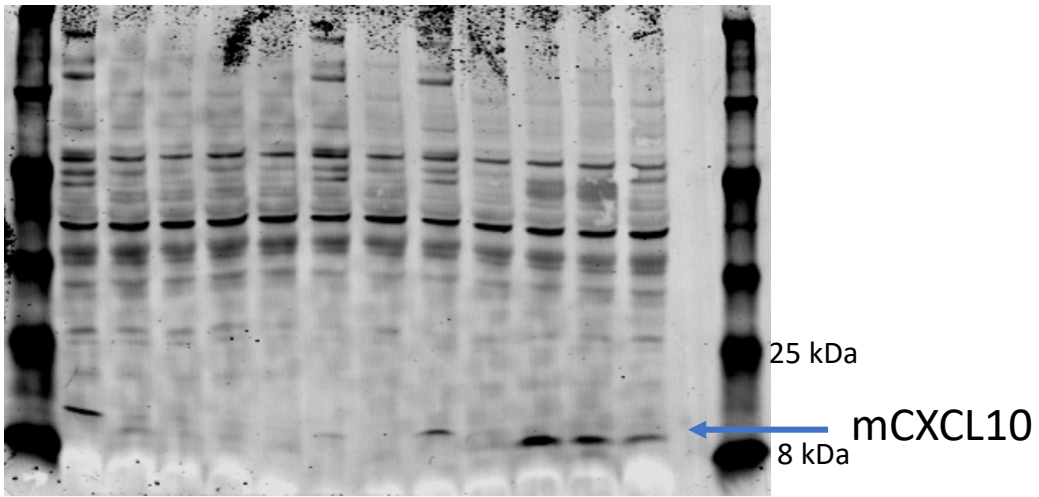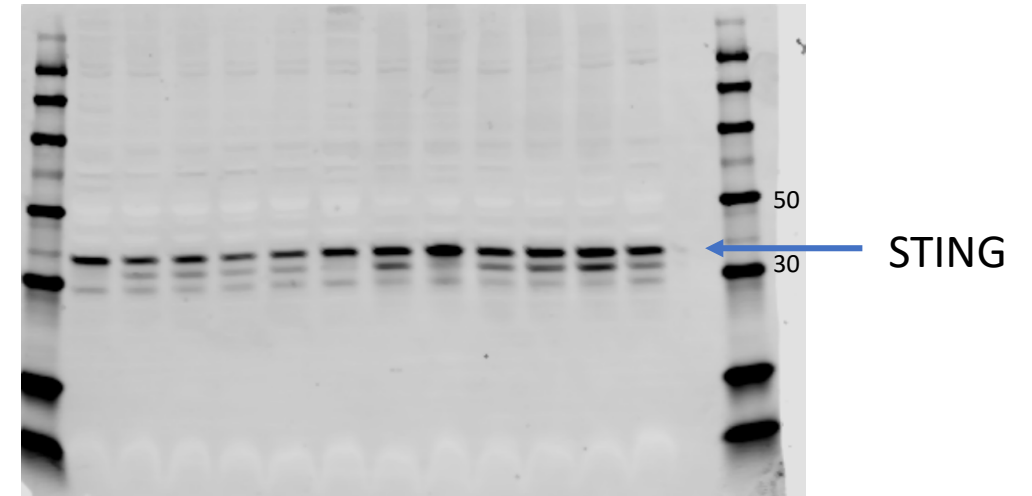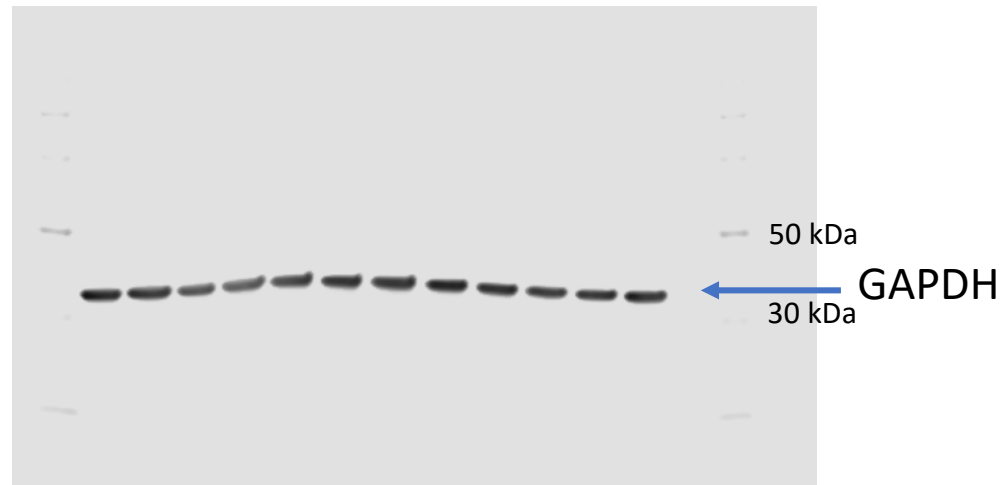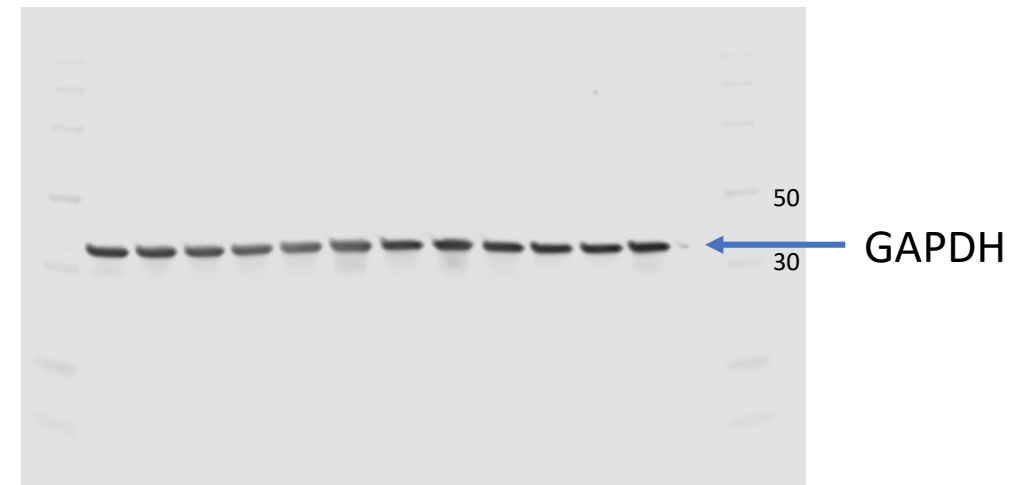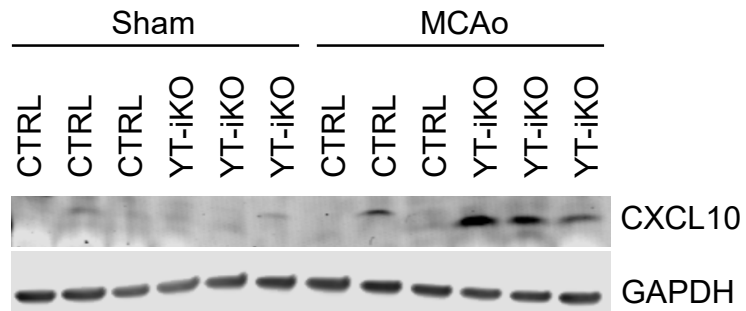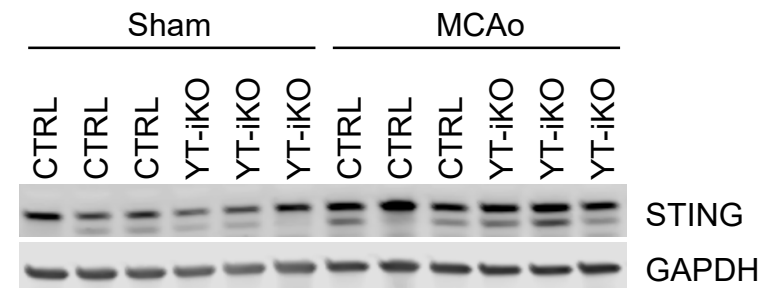

Supplement: Supplementary file 2 — Unedited Gels [file JAH3-15-e040079-s001.pdf]
